# Emerging Trends in Liquid Luminescent Solar Concentrators: Progress and Prospects

**DOI:** 10.1002/smll.202509030

**Published:** 2025-10-30

**Authors:** Mahnoor Hassan, Tofik Ahmed Shifa, Alberto Vomiero, Elisa Moretti, Kassa Belay Ibrahim

**Affiliations:** ^1^ Department of Molecular Sciences and Nano Systems Ca’ Foscari University of Venice Via Torino 155, Mestre Venezia 30172 Italy; ^2^ Division of Materials Science Department of Engineering Sciences and Mathematics Luleå University of Technology Luleå 971 87 Sweden

**Keywords:** chromophores, energy loss mechanisms, liquid LSC, luminescent solar concentrator, phosphorescence

## Abstract

Liquid luminescent solar concentrators (liquid LSCs) have emerged as a promising alternative to conventional solid‐state LSCs for enhancing solar energy harvesting. This review focuses primarily on the two major types of LSCs: liquid‐based and thin‐film‐based systems. By comparing their respective advantages and limitations, it aims to identify how specific performance gaps, such as optical efficiency, quantum yield, scalability, recyclability, ease of fabrication, and cost can be addressed by one type over the other. Particular emphasis is placed on liquid LSCs, which, unlike their solid thin‐film counterparts, offer unique benefits such as solution‐processability, facile large‐area coverage, self‐healing potential, and material reusability. However, despite these benefits, research in this area remains scarce, with relatively few studies published to date. This review aims to provide a comprehensive overview of the recent developments in liquid LSCs, covering fundamental operating principles, key performance metrics, and major advances in luminescent materials, matrix design, and device architectures. Explored potential applications in sustainable energy systems are also reported. This review concludes by discussing ongoing challenges like stability, leakage, and contamination, and presenting future directions highlighting the promise of this underexplored field.

## Graphical abstract

This illustration showcases the design of liquid luminescent solar concentrators highlighting key components such as luminescent materials and waveguide media. It also outlines diverse applications, including building‐integrated photovoltaics, smart windows, photodetectors, agrivoltaics, greenhouse energy systems, and wearable or portable solar devices. The concept emphasizes flexibility, cost‐effectiveness, and integration into sustainable energy technologies.

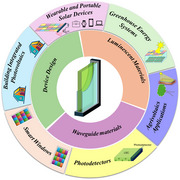

## 1 Introduction

Solar energy is an abundant source of energy that comes directly from the sun's radiation.^[^ ^1^ ^]^ It plays a pivotal role in addressing global energy demands while mitigating environmental concerns associated with fossil fuels, such as greenhouse gas emissions and resource depletion.^[^ ^2^, ^3^, ^4^, ^5^ ^]^ Due to its cost‐effectiveness, it is becoming increasingly popular, and more research has been conducted to capture sunlight and efficiently convert it into usable energy through innovative technologies and materials.^[^ ^6^, ^7^ ^]^ Despite its promise, the widespread adoption of solar energy systems faces challenges, particularly the high initial costs associated with materials, manufacturing, and installation.^[^ ^8^, ^9^, ^10^ ^]^ These economic barriers highlight the need for cost‐effective and efficient solutions to make solar energy more accessible and feasible. Innovations in advanced materials and technologies, such as luminescent solar concentrators (LSCs),^[^ ^11^ ^]^ offer exciting pathways to reduce costs while enhancing energy conversion efficiency. Such advancements are essential for accelerating the global transition to clean energy.^[^ ^12^, ^13^, ^14^, ^15^, ^16^ ^]^

**Figure 1:**
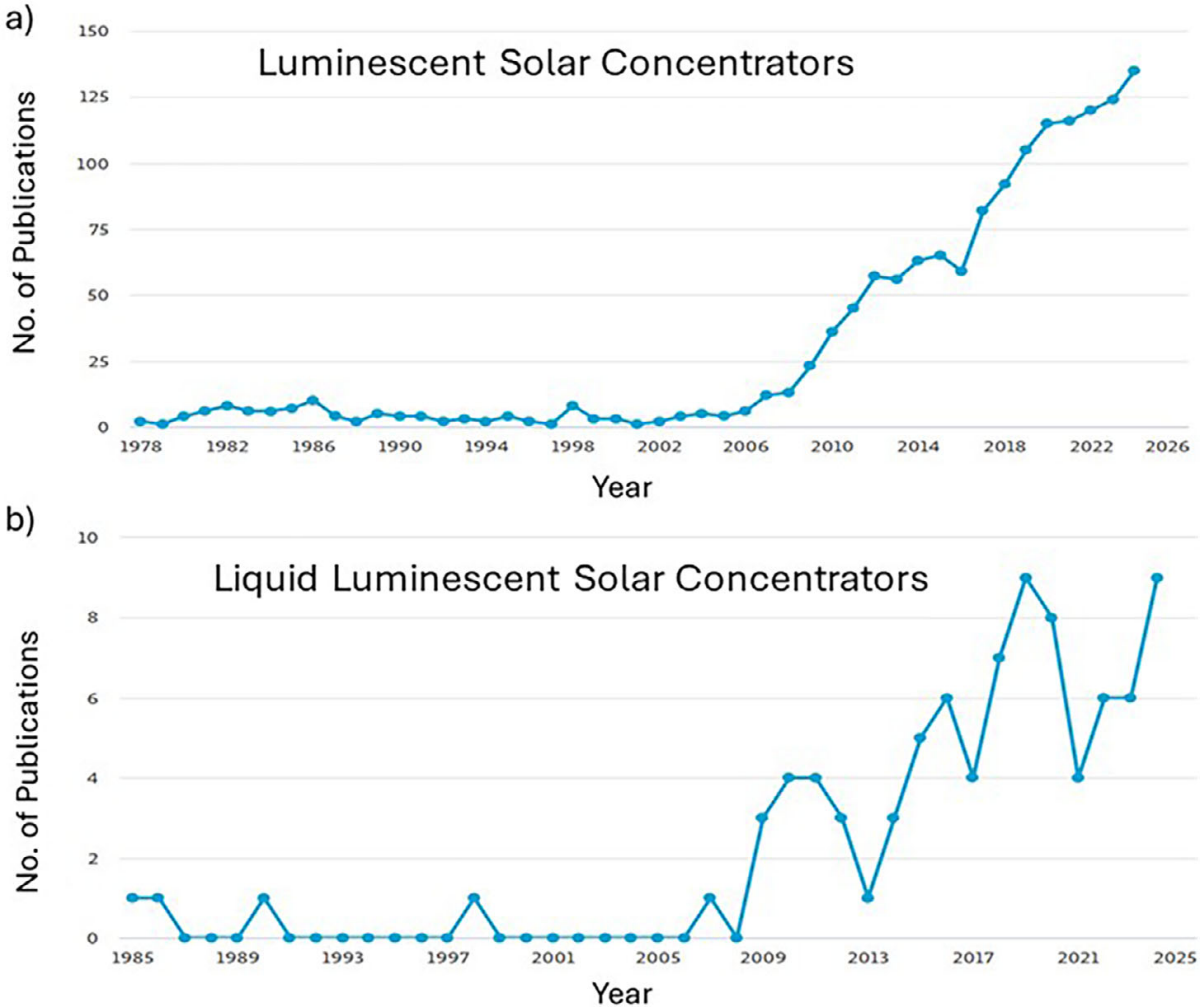
Increasing trend in literature growth: a) number of papers per year published on the topic “Luminescent Solar concentrators” and b) intersection with the topic “Liquid luminescent solar concentrators”. Source “Scopus” based on data from the Scopus database (accessed in April 2025).

LSC is a transparent waveguide designed to capture sunlight and concentrate it at the edges where solar cells are connected to convert the light into electricity.^[^ ^17^ ^]^ These transparent waveguide uses luminescent materials, which can be organic dyes,^[^ ^18^ ^]^ quantum dots (QDs),^[^ ^19^, ^20^, ^21^ ^]^ or rare‐earth elements,^[^ ^22^ ^]^ embedded in a transparent or translucent substrate like glass or polymer.^[^ ^23^ ^]^ These luminescent materials are called luminophores which absorb solar light and re‐emit it at a longer wavelength. The re‐emitted photons are then guided by total internal reflection within the host material, allowing them to reach the thin edge surfaces where solar cells are attached.^[^ ^24^ ^]^ A good LSC can be defined by how efficiently it can transport the emitted light without energy loss.^[^ ^25^ ^]^ LSCs are highly efficient in capturing both direct and diffused sunlight, making them a cost‐effective and versatile solution for solar energy generation, especially in urban or space‐constrained environments.^[^ ^26^, ^27^, ^28^ ^]^ This technology aims to reduce the cost of solar energy by using fewer and smaller solar cells.^[^ ^29^ ^]^ LSCs are cost‐effective due to the use of inexpensive materials such as glass or polymers. In addition, by concentrating the sunlight onto a smaller area, they reduce the amount of photovoltaic (PV) material needed, thereby lowering the overall cost of solar cells. It is a versatile technology as it can be integrated into various applications, such as building‐integrated photovoltaics (BIPVs),^[^ ^30^ ^]^ windows,^[^ ^31^ ^]^ facades,^[^ ^32^ ^]^ and even skylights,^[^ ^33^ ^]^ adding an aesthetic dimension to buildings while generating electricity.^[^ ^34^ ^]^ Their ability to be manufactured in distinct colors and transparency levels allows them to blend seamlessly into architectural designs without compromising functionality. LSCs can be broadly classified into two types based on the nature of their host matrix: liquid LSCs and thin film LSCs^[^ ^35^ ^]^. Thin film LSCs are devices in which luminescent materials, such as dyes, QDs, or carbon dots (CDs)^[^ ^36^, ^37^, ^38^ ^]^ are embedded within a rigid transparent matrix, typically polymers like polymethyl methacrylate (PMMA)^[^ ^39^ ^]^ or polyvinyl pyrrolidine (PVP).^[^ ^35^ ^]^ This system has attracted growing attention from the research community, as evidenced by the substantial and increasing number of scientific publications on LSCs in recent years (**Figure** **1**a). Thin films LSCs are valued for their mechanical robustness, long‐term stability, and ease of integration into building elements such as windows, facades, and rooftops for photovoltaic energy harvesting. However, they face several challenges, including optical losses due to reabsorption and scattering, aggregation of luminophores during fabrication, and limitations in tuning the concentration or replacing degraded materials once solidified.^[^ ^30^, ^40^, ^41^ ^]^ To overcome some of these limitations, liquid‐LSCs have emerged as a promising alternative.^[^ ^42^ ^]^ In these emerging systems, the luminescent materials are dissolved or suspended in a transparent liquid solvent enclosed in a sealed container.^[^ ^43^ ^]^ According to Scopus data in Figure 1b, research on liquid LSCs began more recently and remains a niche area, with relatively few publications despite a gradual rise since 2009, indicating that this promising topic is still underexplored and offers significant room for further research. This configuration allows for better tunability, easier replacement of degraded luminophores, and even potential self‐healing of the system. As a result, liquid LSCs are now increasingly being explored for next‐generation solar harvesting applications, especially where flexibility and adaptability are needed.^[^ ^42^ ^]^

In this review, we present a comprehensive analysis of recent advancements in liquid LSCs and their potential to enhance solar energy conversion. While the existing literature is still relatively limited, the field has demonstrated considerable promise. Recent advances necessitate a comprehensive review and a critical assessment of future prospects. The review begins by outlining the fundamental working principles of typical LSCs, followed by a discussion of key performance metrics used to evaluate their efficiency. The main focus of this review is on recent progress in three crucial areas: the development of luminescent materials, the design of matrix materials, and device architecture tailored specifically for liquid systems. To conclude, this work discusses the current limitations, particularly regarding stability and contamination, and suggests directions for future exploration in this promising field.

## 2 Fundamentals of Luminescent Solar Concentrators

As introduced in Section 1, LSCs operate by absorbing incident sunlight, re‐emitting it at longer wavelengths, and guiding this emission to PV cells positioned at the device edges via total internal reflection. The key components of a typical LSC include luminescent materials (luminophores), a transparent matrix or substrate, photovoltaic cells, and, in some cases, an encapsulation layer depending on the device architecture (e.g., thin‐film or liquid). The transparent matrix, often made of glass or polymer, supports light propagation via total internal reflection.^[^ ^44^ ^]^ PV cells harvest the guided photons, converting them into electricity. The encapsulation layer, when present, serves to protect the system from moisture, UV degradation, and mechanical damage.^[^ ^27^ ^]^

LSCs can be broadly categorized into thin (or thick)‐film and liquid types, based on the nature of the host matrix. In thin‐film LSCs, luminophores are embedded within a rigid, solid polymer,^[^ ^44^ ^]^ which can result in reduced quantum yield and increased reabsorption losses due to immobilization and scattering. In contrast, liquid LSCs suspend luminophores in a fluid medium, which mitigates these losses and allows for enhanced tunability, reusability, and even potential self‐healing capabilities. The most commonly used luminophores include organic dyes, QDs, rare‐earth elements, and CDs, each influencing device performance through their unique photophysical properties.^[^ ^45^ ^]^ In Subsection 2.1, we focus specifically on liquid LSCs, outlining their working principles, benefits over solid‐state counterparts, and recent advancements in materials and device architectures.

### 2.1 Working Principles of Liquid LSCs

**Figure 2:**
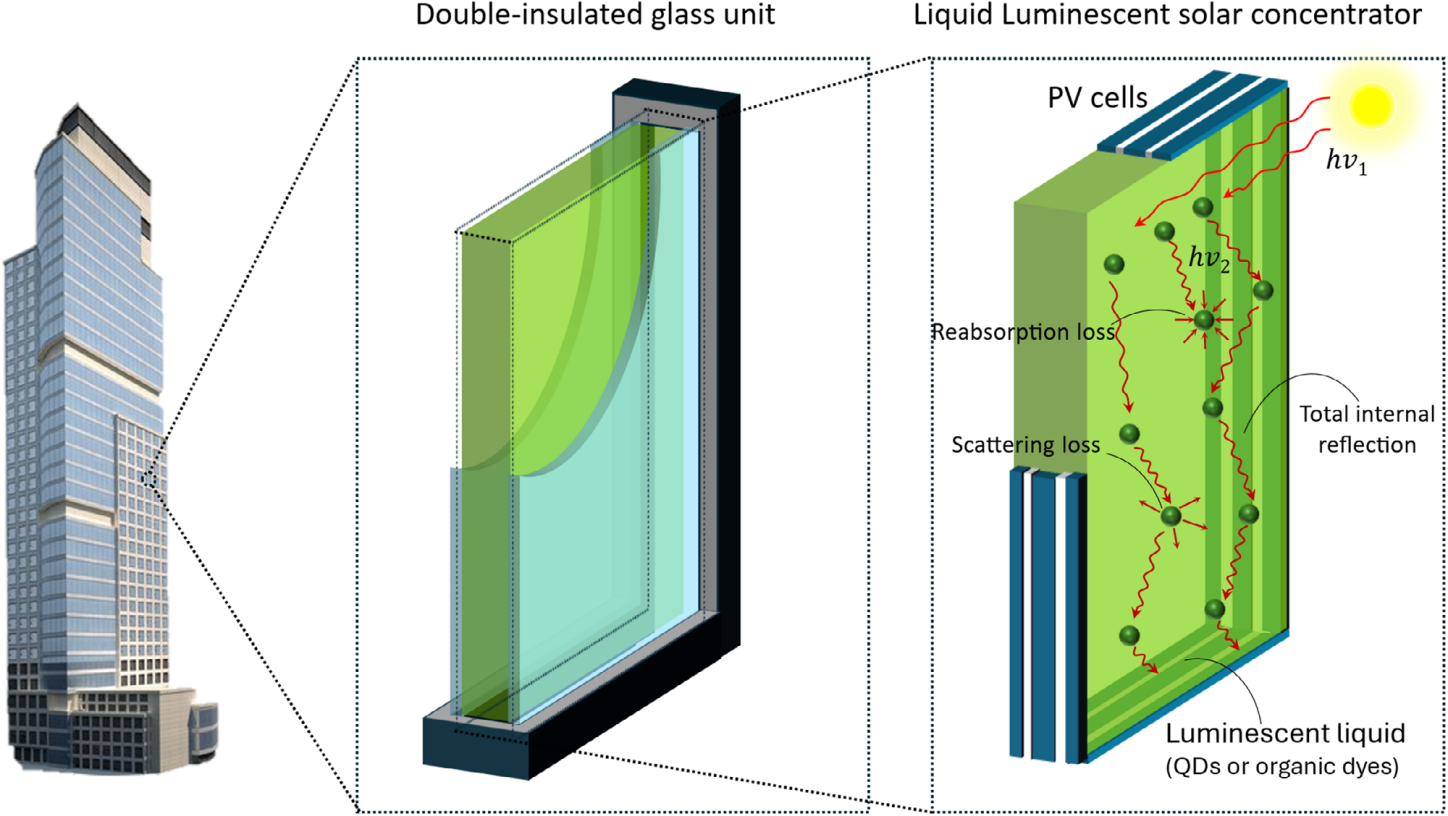
Illustration of the liquid LSCs, where sunlight is absorbed by a luminescent layer, re‐emitted at longer wavelengths, and guided via TIR toward edge‐mounted photovoltaic cells for energy conversion.

LSCs function by utilizing the principles of light absorption, photoluminescence, and waveguiding.^[^ ^46^ ^]^ The process begins with the absorption of sunlight across a broad spectrum by luminescent materials embedded within the transparent matrix. These materials then undergo a transition to an excited state before emitting photons at a longer wavelength due to the Stokes shift, which ensures that emitted light cannot be reabsorbed by the same material, reducing energy losses.^[^ ^47^ ^]^ **Figure** **2** illustrates the working principle of a liquid LSC, a device that captures and concentrates solar energy using a luminescent liquid medium. The system typically consists of a transparent container filled with a fluorescent solution containing luminescent materials such as QDs, CDs, or organic dyes. When sunlight enters the device, these luminescent species absorb part of the incident light and re‐emit it at longer wavelengths. The emitted photons are confined within the liquid‐filled waveguide via total internal reflection (TIR), directing the light toward the device's edges where small PV cells are placed to convert it into electricity. However, some losses can occur due to reabsorption^[^ ^48^ ^]^ but are significantly mitigated in liquid systems by using materials with high photoluminescence quantum yield (PLQY), large Stokes shifts, and maintaining uniform dispersion, as well as optimizing the spatial distribution of the absorbent and emissive materials.^[^ ^15^ ^]^ In addition, the liquid medium allows dynamic control over the concentration and distribution of luminescent species, offering self‐healing and tunability. The waveguide design plays a vital role in maximizing light trapping and reducing angular dependence, enabling efficient solar energy harvesting under diverse lighting conditions. This combination of flexibility, optical efficiency, and ease of material renewal makes liquid LSCs a highly promising approach in next‐generation solar.^[^ ^27^, ^49^, ^50^ ^]^

### 2.2 Luminophore Selection and Challenges

The selection of luminophores plays a crucial role in determining the absorption range, PLQY, and photostability of a liquid LSC system. However, several challenges remain unresolved in optimizing liquid LSC performance. A wide range of luminophores have been investigated for its applications, as reported in several studies.^[^ ^51^, ^52^, ^53^ ^]^ These include traditional luminophores such as organic dyes, QDs, and rare‐earth‐based phosphors. Organic dyes have been widely used due to their high PLQY, tunable optical properties, and ease of processing.^[^ ^54^ ^]^ Recently, a study achieved the highest 𝜂_ext_ values of 7.69% for organic dye‐based LSCs.^[^ ^55^ ^]^ Although organic dyes typically exhibit a small Stokes shift, they offer high PLQY and large absorption coefficients.^[^ ^44^ ^]^ These properties contribute to efficient light harvesting and emission, often leading to superior LSC performance compared to devices utilizing other types of luminophores.^[^ ^42^ ^]^

The use of luminescent organic molecules derived from renewable and natural sources as an alternative to synthetic organic dyes could enhance the sustainability and cost‐effectiveness of the liquid LSCs. This approach preserves key attributes such as synthetic adaptability, strong absorption coefficients, and high emission PLQY. Evidence from previous studies underscores the effectiveness of renewable and naturally sourced materials^[^ ^56^ ^]^ in advancing energy storage solutions, particularly in the development of LSCs.^[^ ^57^ ^]^ Renewable materials suitable for sustainable applications include carbon‐based materials,^[^ ^58^, ^59^, ^60^ ^]^ natural dyes (such as phycobilisomes),^[^ ^61^ ^]^ and silicon‐based materials.^[^ ^27^, ^38^ ^]^ A notable example is the work of Li et al., who synthesized barium‐doped carbon dots (Ba‐CDs) using eco‐friendly precursors, achieving an impressive PLQY of 80.81%.^[^ ^35^ ^]^ Similarly, Gong et al. (2022) developed silicon‐doped carbon dots (Si‐CDs) through a green synthesis approach, attaining a remarkable PLQY of 92.3%.^[^ ^38^ ^]^ These studies highlight the potential of renewable and environmentally friendly materials in advancing luminescent nanomaterials for sustainable applications.

However, organic dyes suffer from intrinsic instability under strong and prolonged solar irradiation, limiting their suitability for long‐term liquid LSC devices. As an alternative class of luminophores, inorganic QDs have gained considerable interest due to their superior photostability, broad absorption spectra, high PLQY, and size‐tunable emission characteristics.^[^ ^62^ ^]^ Typically based on cadmium selenide (CdSe), indium phosphide (InP), or perovskite structures, QDs enable precise control over emission wavelength by adjusting particle size or composition.^[^ ^63^ ^]^ Their narrow emission bands and relatively large Stokes shifts reduce spectral overlap and reabsorption losses,^[^ ^64^ ^]^ making them especially promising candidates for enhancing the efficiency and operational stability of liquid LSCs.

Despite these advantages, conventional QDs based on cadmium or lead raise environmental and regulatory concerns due to their inherent toxicity.^[^ ^43^ ^]^ To address this, recent research has focused on eco‐friendly alternatives, such as CuInS_2_, ZnSe, carbon quantum dots, and silicon nanocrystals, which exhibit lower environmental impact while maintaining desirable optical properties. These materials are particularly attractive for next‐generation liquid LSC's that aim to combine performance with sustainability. To further enhance performance, QDs are often surface‐passivated or engineered into core–shell structures to improve both stability and PLQY.^[^ ^65^ ^]^ These modifications protect the luminescent core from environmental degradation and reduce surface defect states, which would otherwise act as nonradiative recombination centers. Another promising class of luminophores is rare‐earth‐based phosphors, which are known for their excellent photostability, sharp emission peaks, and large Stokes shifts.^[^ ^66^, ^67^ ^]^ While these materials generally have lower PLQY and narrower absorption ranges compared to QDs or organic dyes, their resistance to photobleaching makes them attractive for applications requiring high durability.^[^ ^68^ ^]^

Hence, the selection of luminophore is critical in determining the optical performance, stability, and sustainability of liquid LSCs. A measured balance between spectral properties, environmental safety, and material processability is essential to advancing the field toward scalable, high‐efficiency solar energy solutions.

### 2.3 Functional Mechanisms and Energy Losses

To evaluate the performance of liquid LSCs, it is essential to first establish the general efficiency framework common to all LSCs. The metrics such as geometric gain, optical efficiency, and PV conversion efficiency are widely used in the literature to quantify how effectively these devices capture, guide, and convert sunlight. While these concepts apply broadly to both solid and liquid systems, they are particularly important in liquid LSCs due to the unique characteristics of the liquid medium, such as flexible container geometries, dynamic control of luminophore concentration, and distinctive reabsorption behaviors. Introducing this framework provides a basis for the following discussion of energy losses and efficiency parameters specific to liquid LSCs.

Among the various loss channels, reabsorption plays a primary role in limiting performance. This occurs when photons emitted by one luminophore are reabsorbed by another within the same liquid medium due to overlap between their absorption and emission spectra. To describe how such mechanisms affect performance, the literature commonly employs a set of parameters derived from the same fundamental concepts. The most basic of these is the geometric gain factor (*G*), defined as the ratio between the illuminated surface area of the LSC (top) and the active edge area of the PV cells (edge).

The concentration of light depends on three factors: the geometric gain factor (*G*), the optical efficiency (𝜂_opt_), and the PV cell efficiency (𝜂_PV_). Geometric gain can be expressed by the ratio of area of the concentrator/top to the area of the cell/edge. The relation is typically expressed as:^[^ ^69^ ^]^

(1) $$G=\frac{A_{top}}{A_{edge}}=\frac{A_{concentrator}}{A_{cell}}$$

This geometric factor significantly enhances optical efficiency by increasing the effective light collection area, thus allowing more photons to be funneled into the photovoltaic device. The second factor that can influence the liquid LSCs is optical efficiency, which is primarily related to internal quantum efficiency (𝜂_int_) and external quantum efficiency (𝜂_ext_). The internal efficiency represents the fraction of photons that successfully reach the edges of the LSC (ready to be absorbed by the solar cells) compared to the total number of photons absorbed by the device. Conversely, the external quantum efficiency (EQE) of an LSC is defined as the ratio of the number of photons emitted from the edges of the device (and available for photovoltaic conversion) to the total number of incident photons striking the top surface of the LSC. This metric reflects the overall photon collection and guiding efficiency of the system, accounting for all optical losses, including transmission, reflection, and reabsorption. To further break this down, the absorption efficiency (𝜂_abs_) plays a key role, defined as the ratio of solar flux absorbed (Φ_abs_) to the incoming solar flux (Φ*_i_* ​). Thus, the relationship:^[^ ^70^ ^]^

(2) $$\eta_{opt}=\eta_{ext}=\eta_{abs}\times \eta_{int}$$

This highlights that optical efficiency is inherently tied to the LSC's attributes rather than the photovoltaic cells affixed to it. A comprehensive expression for optical efficiency can be derived through analytical calculations, which yields to the formula:^[^ ^71^ ^]^

(3) $$\eta_{opt}=\eta_{abs}\eta_{int}=\frac{\eta_{abs}\eta_{Trap}\eta_{PLQY}}{1+\beta L\left(\alpha_{abs}+\sigma_{sca}\right)\left[1-\left(\frac{\eta_{PLQY}\alpha_{abs}+\sigma_{sca}}{\alpha_{abs}+\sigma_{sca}}\right)\times \eta_{Trap}\right]}$$

In this equation, various factors come into play: η_*PLQY*_ indicates the PLQY, while η_*abs*_ is the absorption coefficient relevant to the emission wavelength from the LSC nanomaterials. The trapping efficiency of the waveguide is represented by η_*Trap*_, and the scattering coefficient for the emission light is denoted by σ_*sca*_. Here, β is a numerical constant (≈1.4), and *L* signifies the length of the LSC device itself. The optical efficiency can also be represented as electronic optical efficiency ($\eta_{opt}^{el}$) which is most commonly used, and it measures the ratio of the number of detected photons to those incident photons that are detectable, given that PV cells have specific limits on their detection capabilities. When assessing the efficiency of an LSC using calibrated solar PV cells, we can define η_*opt*_ with the following equation:^[^ ^44^ ^]^

(4) $$\eta_{opt}^{el}=\frac{P_{out}}{P_{in}}=\frac{I_{LSC}}{I_{SC}\times G}=\frac{I_{LSC}\times A_{edge}}{I_{SC}\times A_{top}}$$

where *I_LSC_* is the current of the solar cell mounted on the edge of the LSC, *I_SC_* is the current of the solar cell under direct solar simulated illumination, *A_top_* is the top surface area of the LSC, and *A_edge_* is the surface area of the edges mounted on a solar cell. The power conversion efficiency (PCE) can be calculated as follows:^[^ ^14^ ^]^

(5) $$\eta =\frac{J_{sc}\times V_{oc}\times FF}{P_{o}}=\frac{I_{sc}\times V_{oc}\times FF}{P_{o}\times A_{top}}$$

where *V_oc_* is the open circuit voltage, *FF* is the fill factor of the PV cell attached to the edge of LSC, and *P_o_* is the irradiation intensity incident on the surface of LSC. To predict the effect of LSC reabsorption on scalability, two factors can contribute, namely, the quality factor (QF) and the overlap integral (OI). The QF is a dimensionless parameter that quantifies the performance of photonic systems, particularly regarding their efficiency in light absorption and emission. It can be expressed as:^[^ ^72^ ^]^

(6) $$QF=\frac{\alpha_{1}}{\alpha_{2}}$$

In Equation (5), α_1_ represents the absorption coefficient of a specific material or layer, typically the active component that absorbs the incoming light, while α_2_ denotes the absorption coefficient of another material or layer, often a passive component that can influence the overall light management of the system. By taking the ratio of these two coefficients, QF provides insight into the relative absorption efficiency of the active component compared to the passive one. A higher QF indicates that the active material is more effective at absorbing light relative to the passive material, suggesting improved performance in applications such as LSCs or other optical devices where efficient light harvesting is essential.  The OI is defined by the following equation:^[^ ^73^, ^74^ ^]^

(7) $$OI=\int_{0}^{\infty }A\left(\lambda \right)PL^{*}\left(\lambda \right)d\lambda$$

where *A*(λ) is the absorption spectrum and PL*(λ) denoted the PL emission spectrum. This integral quantifies the degree to which the absorption characteristics of the material overlap with its emission characteristics across all wavelengths, thus providing insight into the material's ability to efficiently convert absorbed light into emitted light. A larger overlap integral indicates a stronger interaction between the absorbed and emitted wavelengths, signifying better performance in applications such as LSCs. In contrast, the normalized overlap integral OI* is calculated using the formula:^[^ ^73^, ^74^ ^]^

(8) $$OI^{*}=\frac{\int_{0}^{\infty }A\left(\lambda \right)PL^{*}\left(\lambda \right)d\lambda }{\int_{0}^{\infty }PL^{*}\left(\lambda \right)d\lambda }$$

where the numerator remains the same as in the overlap integral, while the denominator calculates the total emitted light across all wavelengths. This dimensionless value allows for comparisons between different materials and devices without being affected by the intensity of the light. However, it is also essential to consider how the reflectance influences the absorption and emission properties of these luminescent materials. For instance, when evaluating devices such as LSCs, the reflection coefficient (*R*) of the surface is calculated as:^[^ ^28^ ^]^

(9) $$R={\left|\frac{n_{wg}-n_{s}}{n_{wg}+n_{s}}\right|}^{2}$$

**Figure 3:**
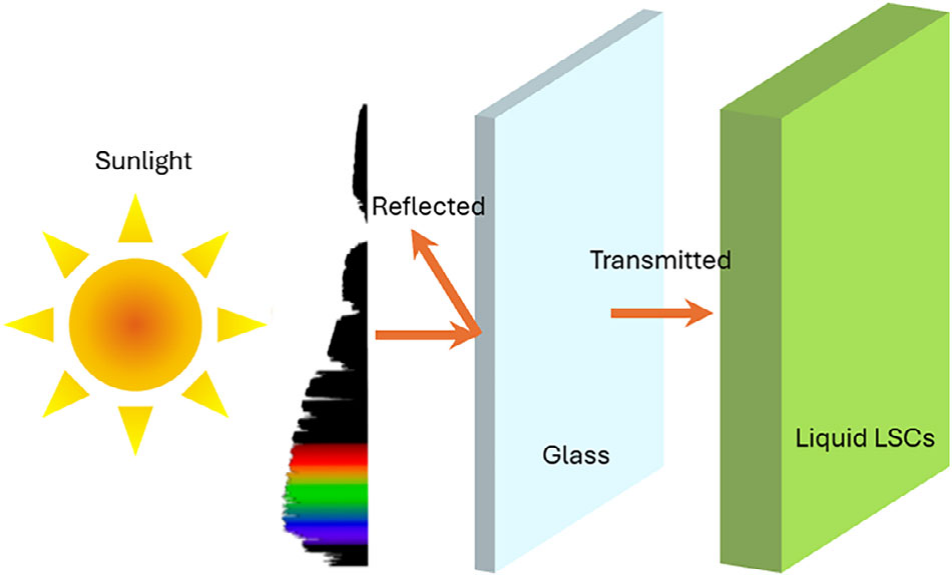
Illustration showing the interaction of sunlight at the glass–liquid interface of an LSC. A portion of the incident sunlight is reflected at the surface, while the rest is transmitted into the luminescent medium for absorption.

Here, *n_wg_* is the refractive index of the waveguide, and *n_s_* is the refractive index of the surrounding medium. This equation describes the fraction of incident light that is reflected back rather than transmitted into the material. When sunlight strikes the surface of the LSC, any light that is reflected instead of absorbed reduces the overall efficiency of the system. Hence, managing the reflectance at the interface (see **Figure** **3**) is crucial to ensuring that as much light as possible enters the luminescent material for effective absorption and subsequent photoluminescent emission, ultimately enhancing the device's performance in converting sunlight into usable energy.

### 2.4 Reabsorption Mitigation Strategies

One of the dominant mechanisms contributing to optical losses in LSCs (liquid or thin film) is reabsorption. It is the process by which photons emitted by a luminophore are reabsorbed by neighboring emitters due to spectral overlap. This parasitic absorption shortens the effective photon path to the edge‐mounted PV cells, thereby limiting η_opt_ and reducing the overall PCE. A range of strategies have been developed to suppress reabsorption by either modifying the photophysical characteristics of luminophores or by engineering the spatial geometry and internal architecture of the LSC system.

De Clercq et al. in his study designed a self‐assembling copolymer matrix consisting of a 1:1 mixture of PMMA and polystyrene‐*block*‐poly(acrylic acid) (PS‐*b*‐PAA). Upon solvent evaporation, the system undergoes microphase separation to form micrometer‐sized hollow domains (≈1.6 µm) within the LSC matrix. These voids reduce the optical density of the luminescent region without altering its footprint, effectively lowering the probability of photon reabsorption. The authors observed a significant reduction in the average number of reabsorption events (from 4.0 to 1.8) and an increase in PLQY from 77% to 87%. This exemplifies how internal void geometry in polymer matrices can passively control photon paths and minimize reabsorption.^[^ ^75^ ^]^

From a materials perspective, Liao et al. tackled the problem by employing cesium copper halide nanocrystals (Cs_3_Cu_2_X_5_), which exhibit broadband emission from self‐trapped excitons (STEs) with extremely large Stokes shifts. Unlike typical QDs that suffer from emission‐absorption overlap, these nanocrystals showed almost no spectral overlap, enabling a reabsorption‐free behavior even in relatively thick waveguides. This material architecture achieved edge optical efficiencies of 70% and PCEs of 1.8% in 8 × 8 × 0.5 cm^3^ devices, demonstrating scalability and high efficiency without reliance on heavy metals.^[^ ^76^ ^]^

In another geometry‐dependent strategy, Gu et al. utilized Mn^2^⁺‐doped perovskite QDs (CsPbCl_3_:Mn) to shift emission from the host band edge (≈410 nm) to a deeper Mn^2^⁺‐centered emission at ≈620 nm. This doping‐induced spectral separation ensures that emitted photons lie largely outside the absorption window of the host lattice. Because the Mn^2^⁺ centers are embedded within the crystal lattice, their emission is spatially decoupled from absorption centers. As a result, the optical efficiency of the luminescent solar concentrator increased from 1.63% to 2.23%, representing an ≈37% improvement.^[^ ^77^ ^]^

Wilson et al. provided one of the earliest comprehensive analyses of reabsorption losses in LSCs, combining experimental measurements with ray‐tracing simulations to directly quantify the effect of spectral overlap on device performance. By comparing a high quantum yield organic dye (Lumogen F Red 305, PLQY ≈100%) with a lanthanide‐based luminophore possessing a significantly larger Stokes shift (PLQY ≈86%), the authors demonstrated that reduced reabsorption can outweigh even superior PLQY in determining overall efficiency. Despite the lower intrinsic quantum yield, the lanthanide complex yielded a higher optical efficiency (η_opt_) of 64% compared to 50% for the organic dye, primarily due to its minimized spectral overlap. These findings underscore that high PLQY alone is not sufficient; spectral separation plays a critical role in mitigating reabsorption and enhancing photon transport within the waveguide.^[^ ^78^ ^]^

Gong et al. addressed reabsorption losses in LSCs by engineering Si‐CDs with a large Stokes shift and high PLQY. The Si‐CDs exhibited a PLQY of 92.3% in solution and 80.5% when embedded in a PVP matrix, ensuring high radiative efficiency. To quantify reabsorption, the authors calculated the OI, a metric representing the spectral overlap between absorption and emission. As the concentration of Si‐CDs increased, the OI also rose from 6.88 at 0.15% to 9.31 at 0.20%, and reaching 10.59 at 0.30%, indicating increased reabsorption potential at higher loadings. Despite this, the optimal concentration (0.20%) delivered the highest device performance with a PCE of 4.36% for a 5 × 5 cm^2^ LSC and 2.06% for a 15 × 15 cm^2^ device under natural sunlight (35 mW cm^−2^), demonstrating a practical trade‐off between concentration, reabsorption, and overall efficiency.^[^ ^38^ ^]^

Taken together, the studies discussed provide a comprehensive roadmap for mitigating reabsorption losses in LSC technology. The body of work demonstrates that an effective solution requires a dual approach: careful manipulation of both the photophysical properties of the luminophore (e.g., maximizing Stokes shift) and the internal geometry of the LSC matrix (e.g., passive control via voids). By strategically balancing these factors, researchers can significantly improve the efficiency of liquid LSCs as well.

## 3 Recent Advancements in Liquid LSCs

Recent developments in Liquid LSCs have focused on improving the efficiency, stability, and versatility of the system by optimizing the luminescent materials, matrix materials, and device design. These advancements are driven by the need to enhance performance, reduce costs, and explore new applications for LSCs in various fields like flexible electronics and portable solar devices.

### 3.1 Luminescent Materials

**Figure 4:**
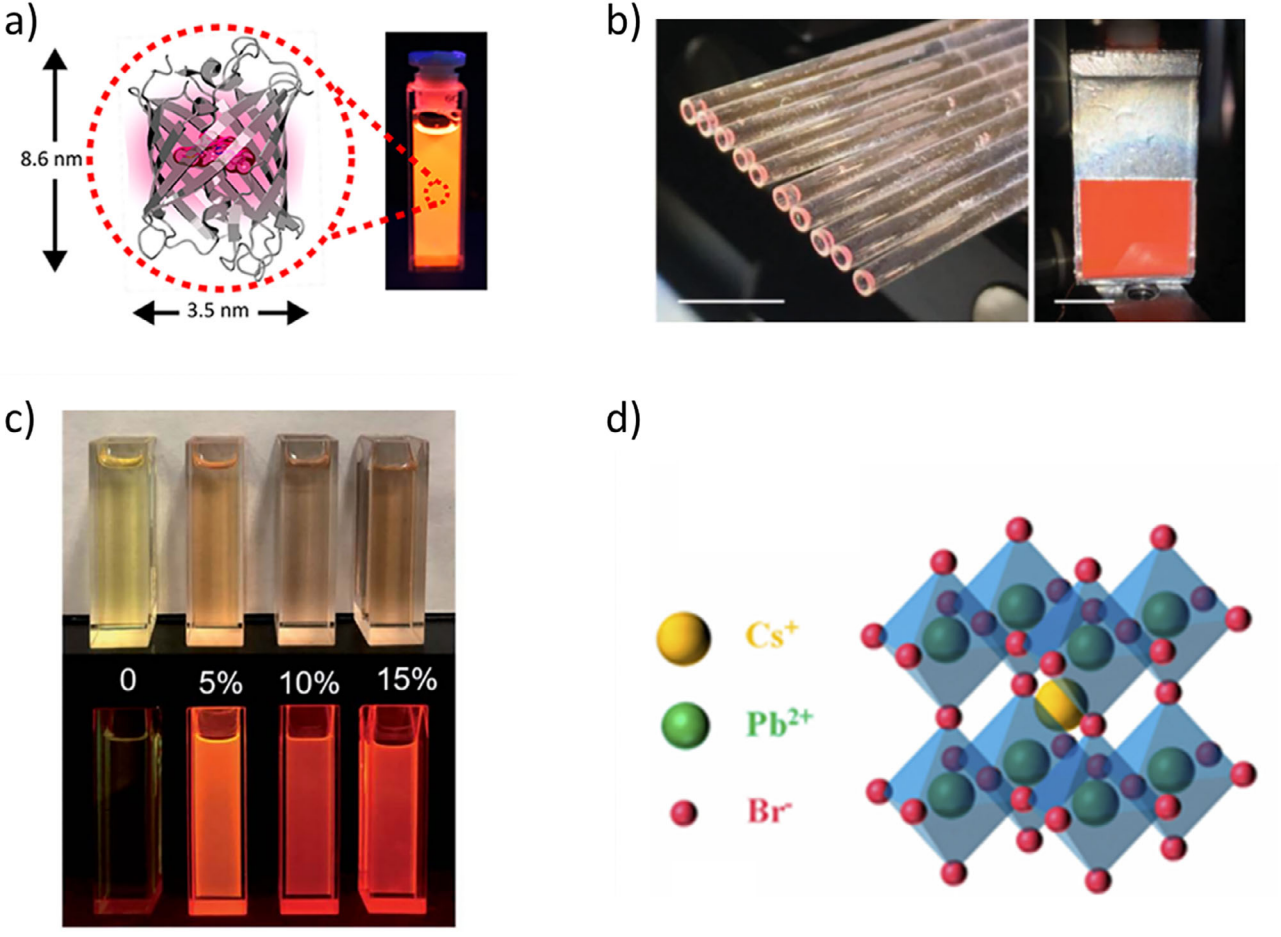
a) A schematic of the mScarlet fluorescent protein, which is water‐dispersible and emits fluorescence under UV light. Reproduced (Adapted) with permission.^[^ ^79^ ^]^ Copyright 2019, ACS. b) Bundle of cylindrical LSCs (c‐LSC) alongside a planar LSC (p‐LSC) enhanced with reflective tape. Reproduced (Adapted) with permission.^[^ ^42^ ^]^ Copyright 2019, Wiley. c) Photographs of synthesized QDs with different Cu doping levels (Cu/Zn–In ratios) under ambient light and UV illumination. Reproduced (Adapted) with permission.^[^ ^43^ ^]^ Copyright 2020, RSC. d) The three‐dimensional crystal structure of CsPbBr_3_ QDs. Although a solid‐state lattice, these nanocrystals are widely employed as luminophores dispersed in liquid hosts due to their high PLQY and tunable emission. Reproduced (Adapted) with permission.^[^ ^83^ ^]^ Copyright 2022, Optica.

**Figure 5:**
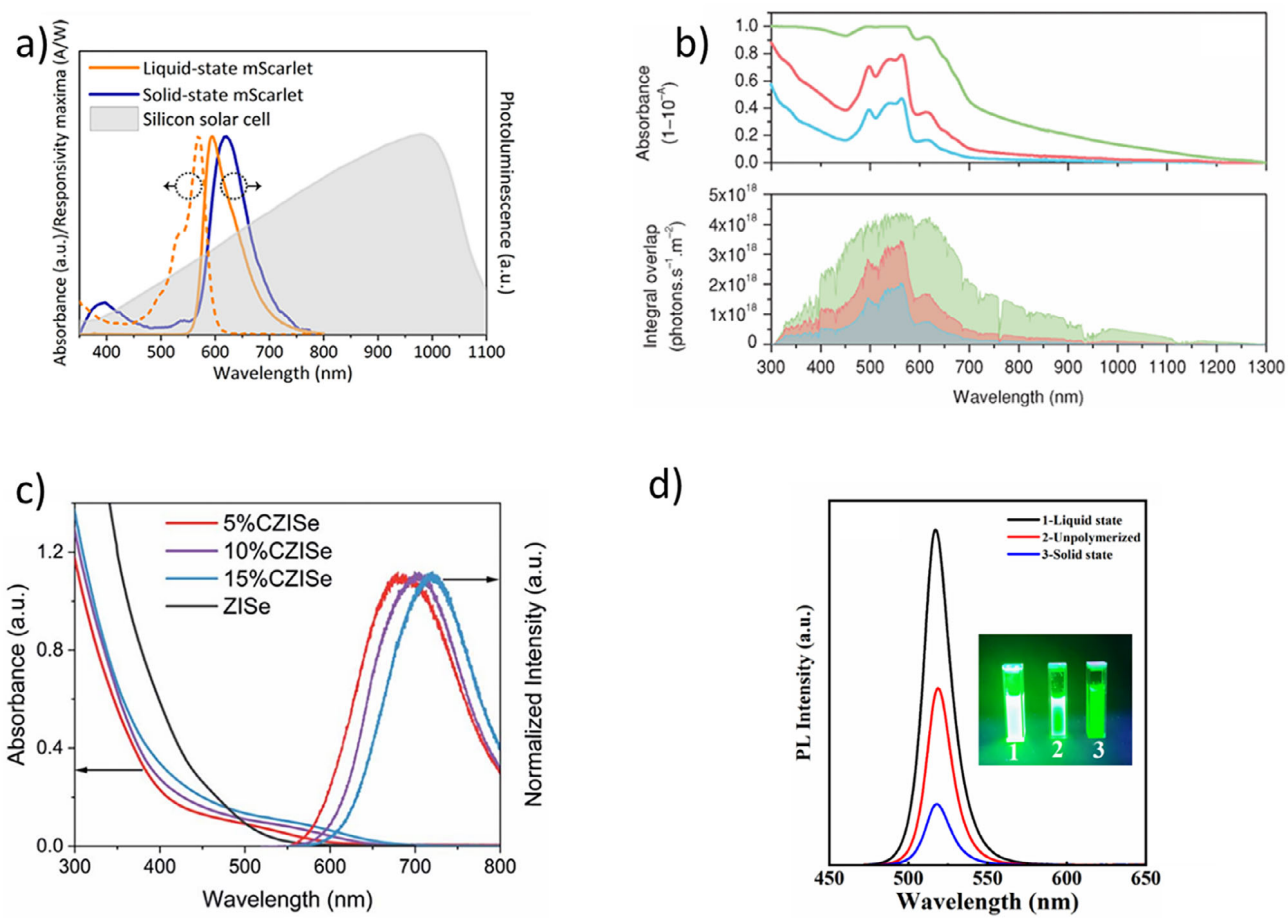
a) Normalized absorbance and PL spectra of liquid mScarlet, solid‐state PL, and the silicon solar cell responsivity (gray area). Reproduced (Adapted) with permission.^[^ ^79^ ^]^ Copyright 2019, ACS. b) Absolute absorbance at 1.7 × 10^−7^ m (blue), 3.3 × 10^−7^ m (red), and 17 × 10^−7^ m (green), with integrated overlap with the solar photon flux. Reproduced (Adapted) with permission.^[^ ^42^ ^]^ Copyright 2019, Wiley. c) Absorption and PL emission spectra of ZISe and CZISe QDs. Reproduced (Adapted) with permission.^[^ ^43^ ^]^ Copyright 2020, RSC. d) PL spectra of CsPbBr_3_ QDs in *n*‐hexane (black, No. 1–Liquid), in OSTE without photo‐initiator (red, No. 2–Unpolymerized), and in OSTE after polymerization with photo‐initiator (blue, No. 3–Solid). Reproduced (Adapted) with permission.^[^ ^85^ ^]^ Copyright 2022, Optica.

The choice of luminescent materials is crucial for the performance of liquid‐state LSCs. Recent advancements have focused on developing more efficient and stable luminescent materials that can provide higher PLQY and minimize reabsorption losses. QDs have emerged as a key material due to their tunable absorption and emission properties, high PLQY, and narrow emission spectra, which reduce the overlap with their absorption spectra and minimize reabsorption. **Table** **1** summarizes the key materials and their corresponding optical and efficiency parameters reported in studies of liquid LSCs. Furthermore, new organic dyes with enhanced stability and efficiency have been developed, along with doped nanoparticles (e.g., doped with metals like silver or gold) to increase photonic emission and minimize photobleaching over time. Perovskite‐based luminescent materials are also being explored for their high efficiency and ease of fabrication, though issues related to long‐term stability under environmental conditions remain a challenge. The combination of these advanced materials aims to improve the overall light‐harvesting capabilities and longevity of liquid LSCs. In the literature of liquid LSCs, a study has reported the use of fluorescent proteins in LSCs. Specifically, mScarlet fluorescent proteins in *Escherichia coli* expression system,^[^ ^79^ ^]^ which turns out to be in red spectral region, with most intense emission, and a PLQY of 61%. mScarlet, like other fluorescent proteins, encapsulates a chromophore within a nanocylinder ≈8.6 nm long and 3.5 nm wide (**Figure** **4**a). mScarlet fluorescent proteins were characterized in both liquid and solid forms to evaluate their optical behavior. In the liquid state, they exhibited an absorption peak at 569 nm and an emission peak at 595 nm.^[^ ^79^ ^]^ Upon transitioning to the solid state, the emission peak shifted to 620 nm, indicating a redshift in photoluminescence (**Figure** **5**a). Förster‐type resonance energy transfer may occur as the transition dipole moments come into close proximity and begin to interact.^[^ ^80^ ^]^ Figure 5a can also help in determining the photoluminescence spectral profile of proteins. In Table 1, reported efficiency values reflect those given in the original studies and include different metrics such as optical conversion efficiency, external photon efficiency, or external quantum efficiency, often measured under different illumination conditions (AM1.5G solar simulator, LED excitation, planar versus cylindrical geometry). As highlighted in the LSC consensus statement (Yang et al., Joule, 2022),^[^ ^81^ ^]^ such values are not directly comparable across studies. They are presented here only as representative examples of liquid‐phase luminophore performance. Unlike solid‐state LSCs, where Lumogen F Red 305 in PMMA serves as a community benchmark, liquid LSCs currently lack a standardized benchmark material or protocol, underscoring the need for harmonized testing and reporting.

**Table 1:** Materials and key photophysical properties of materials used for liquid LSCs.

Materials	Abs and Em range	PLQY [%]	Reported efficiency (metric type)	Refs.
mScarlet fluorescent proteins	Abs: 569 nm, Em: 595 nm	≈61	External LSC efficiency of 2.58%; up to ≈5% at high concentration	[79]
R‐phycoerythrin (R‐PE)	Emission with two components, 577 and 632 nm	≈39	Optical conversion efficiency values up to 6.88% (planar), 4.74% (cylindrical)	[42]
Cu‐doped ZnInSe QDs	Abs: 620–680 nm, Em: 677–718 nm	≈63	Optical efficiency of over 3.5%	[43]
CsPbBr_3_ QDs	Abs: 300–510 nm, Em: 517 nm	≈89	External quantum efficiency up to 13.44%; optical efficiency 2.32%	[82]
Ba‐CDs	Abs: 410 nm, Em: 525 nm	≈81	Not measured in the liquid phase	[35]

Another study on fluorescent proteins, conducted in 2019, utilized R‐phycoerythrin (R‐PE) extracted from *Gracilaria* sp. algae.^[^ ^42^ ^]^ In this study, an aqueous solution of the protein was used to fill hollow‐core cylindrical optical fibers. The R‐PE fluorescent protein, with strong absorption in the UV–visible region (300–550 nm), re‐emits light in the red spectral range (550–700 nm), achieving a PLQY of around 39%. In this work, liquid LSCs were fabricated in various shapes, including planar and cylindrical forms, as seen in Figure 4b. The resulting devices achieved impressive optical conversion efficiencies of 6.88% for the planar configuration and 4.74% for the cylindrical bundle. The absorption spectra of three selected samples are shown in Figure 5b. No significant spectral shifts were observed with varying concentrations; however, the absorption intensity increased in proportion to concentration. A noticeable component appears in the 590–690 nm range, which may be attributed to the presence of additional phycobiliproteins, such as phycocyanin and allophycocyanin.^[^ ^61^ ^]^ The overlap integral between the absorption spectrum of R‐PE and the solar irradiance was calculated to quantify the LSCs’ capacity for sunlight absorption and photovoltaic conversion by using the following equation:^[^ ^82^ ^]^

(10) $$O=\int_{\lambda_{1}}^{\lambda_{2}}N_{AM1.5G}\left(\lambda \right)\times \left(1-10^{-A\left(\lambda \right)}\right)d\lambda$$

The O values increase with the concentration as seen in Figure 5b. Based on the maximum calculated overlap integral, the 17 × 10^−7^ m aqueous solution is capable of absorbing around 27% of the solar photon flux reaching Earth's surface, estimated at 4.3 × 10^21^ photons s^−1^ m^−2^.

A study also explored the development of environmentally friendly luminophores by synthesizing Cu‐doped ZnInSe QDs,^[^ ^43^ ^]^ a type of green transition metal‐doped semiconductor (Figure 4c). The incorporation of copper enabled precise modulation of photoluminescent emissions across the visible spectrum and significantly broadened the Stokes shift. As a result, the QDs achieved a PLQY of up to 63%. Furthermore, performance evaluations comparing polymer‐based and liquid‐based LSC architectures revealed that, with optimized conditions, liquid QD‐based systems free of heavy metals could attain optical conversion efficiencies exceeding 3.5%. These findings underscore the promise of sustainable QD materials in advancing LSC technologies. A significant Stokes shift is evident in Figure 5c, potentially resulting from multi‐excitonic recombination behavior inherent to I–III–VI group nanocrystals.^[^ ^84^ ^]^ In addition to doped quantum dots, halide perovskite nanocrystals such as CsPbBr_3_ have recently emerged as promising luminophores for liquid LSCs. Li et al. demonstrated CsPbBr_3_ QDs injected into a self‐assembled quartz glass mold, evaluating their performance both in liquid hosts such as n‐hexane and in hybrid solidifying matrices like off‐stoichiometric thiolene (OSTE) polymers.^[^ ^85^ ^]^ Figure 4d shows the crystal lattice of CsPbBr_3_, which underpins their high PLQY and tunable bandgap. Although the figure depicts the solid‐state structure, in practice these QDs are dispersed in solvents or ionic liquids for liquid LSCs, where their optical tunability and emission stability are critical to device performance. In Figure 5d, three different QDs can be seen: CsPbBr_3_ QDs (1) in *n*‐hexane, (2) in OSTE without photo‐initiator, and (3) in OSTE after polymerization with photo‐initiator. Weaker green emission from the QDs/OSTE composites, under 365 nm UV light, is observed in comparison to pure QDs. There is also a decline in PL intensity, which is likely a result of poor QD‐polymer compatibility, leading to partial degradation of the QDs. The PLQY of QDs decreases markedly upon incorporation into the OSTE polymer, attributed to polymerization‐induced phase transitions. As a result, liquid LSCs are preferred for higher PL performance. The fabricated devices exhibited an internal quantum efficiency of 32% and an external quantum efficiency of 13.44%. The position and shape of the emission spectral profiles remain unchanged in three selected samples, namely CsPbBr_3_ QDs distributed in *n*‐hexane (QDs solution), OSTE without photo‐initiator as well as in OSTE after polymerization with photo‐initiator, respectively (Figure 5d). These results imply that the polymerization process preserves the surface integrity of the QDs, avoiding the formation of defect states that could otherwise facilitate nonradiative decay pathways.^[^ ^43^ ^]^

In the field of liquid LSCs, a variety of non‐toxic, environmentally friendly luminescent materials such as CDs and metal‐free organic fluorophores remain underexplored despite their promising optical properties. These green alternatives offer a safer and more sustainable option compared to conventional toxic dyes or QDs. Moreover, since liquid LSCs do not rely on a solid polymer matrix, issues related to long‐term durability, reabsorption and photodegradation are significantly reduced, further enhancing their potential for high‐performance solar harvesting systems.^[^ ^43^, ^86^ ^]^

### 3.2 Matrix Materials (Waveguide Materials)

In liquid‐state LSCs, the matrix material plays a vital role in suspending and stabilizing the luminescent materials. Recent advancements in matrix materials focus on improving the stability, viscosity, and refractive index of the liquid medium. The matrix must be transparent, with a refractive index matching the waveguide to facilitate total internal reflection and efficient light trapping. Common matrix materials include water, which is ideal for water‐soluble luminescent materials like CDs, while ethanol and methanol are frequently used for organic dyes or QDs with better solubility in alcohols. Glycols such as ethylene glycol and glycerol offer higher viscosity, reducing convective currents and enhancing stability. Ionic liquids provide excellent thermal and chemical stability with tunable optical properties, making them promising alternatives. In addition, nonpolar solvents like silicone oils or mineral oils are suitable for hydrophobic QDs or dyes, preventing aggregation and improving long‐term performance (**Table** **2**). For instance, Li et al. investigated the use of CsPbBr_3_ QDs in liquid LSCs, employing *n*‐hexane as the dispersion medium. As a nonpolar organic solvent within the oil‐based media category, n‐hexane offers advantages such as excellent solubility for hydrophobic QDs, reducing aggregation and preserving their optical properties. In addition, they conducted a comparative study using CsPbBr_3_ QDs dispersed in both n‐hexane and an OSTE polymer. The results revealed that, unlike the QD solution in n‐hexane, the PLQY of the QDs/OSTE composite significantly declined due to phase transitions induced by polymerization, highlighting the impact of matrix selection on luminescent efficiency.^[^ ^85^ ^]^

**Table 2:** Quantitative physical and optical properties of common matrix materials used in liquid and solid LSCs.

Matrix material	Refractive index (*n*, 589 nm)	Viscosity (η, 25 °C, mPa s)	Thermal stability (°C)	Visible transmission (400–700 nm)	Refs.
Water	1.333	0.89	100 (bp)	>95% (thin layer)	[97, 98]
Ethanol	1.361	1.07	78 (bp)	>90%	[98, 99, 102]
Methanol	1.328	0.54	65 (bp)	>90%	[98, 99, 103]
Ethylene glycol	1.431	16.1	197 (bp)	>90%	[98, 100]
Glycerol	1.473	945	290 (bp)	>90%	[98, 100, 101]
n‐Hexane	1.375	0.31	69 (bp)	>90%	[102, 103]
Ionic liquids (e.g., [BMIM][BF_4_])	1.40–1.55	100–400	>300 (TGA onset)	High (>90%)	[104, 105]
PMMA (solid)	1.489	–	*T*_g_ ≈ 105; stable >200	≈92% (3 mm slab)	[106]
Fused silica (quartz)	1.458	–	>1000	>90% (1–10 mm)	[107, 108]
Silicone oils (PDMS)	1.403	100–1000 (grade dependent)	Stable to ≈300	>90%	[109]

Polymer‐based gels and silica sol‐gels have also gained attention because of their ability to provide both stability and flexibility, preventing the dispersion of luminescent materials. In some cases, ionic liquids have been explored for their low volatility, high optical clarity, and ability to suspend nanoparticles without aggregation. Researchers are also working on hybrid matrices, which combine liquids and solid components to achieve a balance between flexibility and stability. These matrix materials are being engineered to ensure that they can hold the luminescent materials in a stable dispersion while maintaining optical clarity and minimizing losses due to scattering or absorption.

An important consideration in the development of liquid LSCs is the selection of a solvent that is both optically transparent and chemically stable under prolonged exposure to sunlight. The solvent functions as the medium for dispersing the luminescent materials, and its transparency is crucial to allow efficient light absorption and waveguiding within the device. Any optical absorption by the solvent in the relevant spectral range can significantly hinder device performance. Moreover, photochemical stability is essential to prevent degradation, photobleaching, or the formation of by‐products that could quench luminescence or compromise the clarity of the system. Therefore, choosing a transparent, sunlight‐stable solvent is fundamental to ensuring the optical efficiency, durability, and long‐term functionality of liquid LSCs.

### 3.3 Quantitative Comparison of Host Matrix Properties

The previous discussion highlights the qualitative importance of various matrix materials in liquid LSCs. However, a deeper understanding of their performance requires a quantitative comparison of key physical properties. Table 2 provides a comprehensive list of these characteristics, spanning a wide index viscosity–stability space.

Low‐refractive‐index (*n*), low‐viscosity (η) hosts like ethanol and methanol are easy to process but suffer from high volatility. In contrast, glycols and silicone oils offer higher refractive indices and much greater viscosities, which effectively suppress convective mixing and prevent nanoparticle aggregation. Ionic liquids combine non‐volatility with tunable refractive index and exceptional thermal stability (>300 °C), though they often have higher viscosities.

For solid hosts, PMMA and fused silica provide the highest refractive indices among common materials (≈1.49 and 1.458, respectively) with excellent visible transmission. This enables efficient waveguiding but necessitates careful consideration of UV durability for PMMA or the rigid form factor of silica. These explicit, quantitative comparisons make the inherent trade‐offs clear and serve as a guide for selecting the optimal host material to achieve desired efficiency and long‐term stability in LSC/LLSC devices.

### 3.4 Device Architecture and Design Strategies

Advancements in device design and fabrication are crucial for improving the performance and scalability of liquid LSCs. One area of focus has been on optimizing the waveguide geometry to maximize light trapping and minimize energy losses. Researchers are developing more robust encapsulation techniques to prevent leakage and ensure the long‐term stability of liquid LSCs. Innovations in flexible substrates allow for the integration of LSCs into portable devices and flexible electronics, opening up new possibilities for applications such as solar‐powered wearables and BIPVs. The fabrication processes for liquid LSCs have also improved, with advances in nanofabrication and layering techniques that allow for precise control over the dispersion of luminescent materials and the uniformity of the matrix. Moreover, new methods for sealing and encapsulating liquid‐based systems have been developed to prevent evaporation and ensure that the device remains functional over extended periods, even under varying environmental conditions. For example, to evaluate solvent evaporation, a liquid LSC (20 × 20 × 2 mm) was kept at 50 °C for 4 h, resulting in an ≈5% mass loss due to a 1 mm injection hole. When the hole was sealed with tape, no significant mass loss was observed, indicating good solvent stability under thermal stress.^[^ ^43^ ^]^

**Figure 6:**
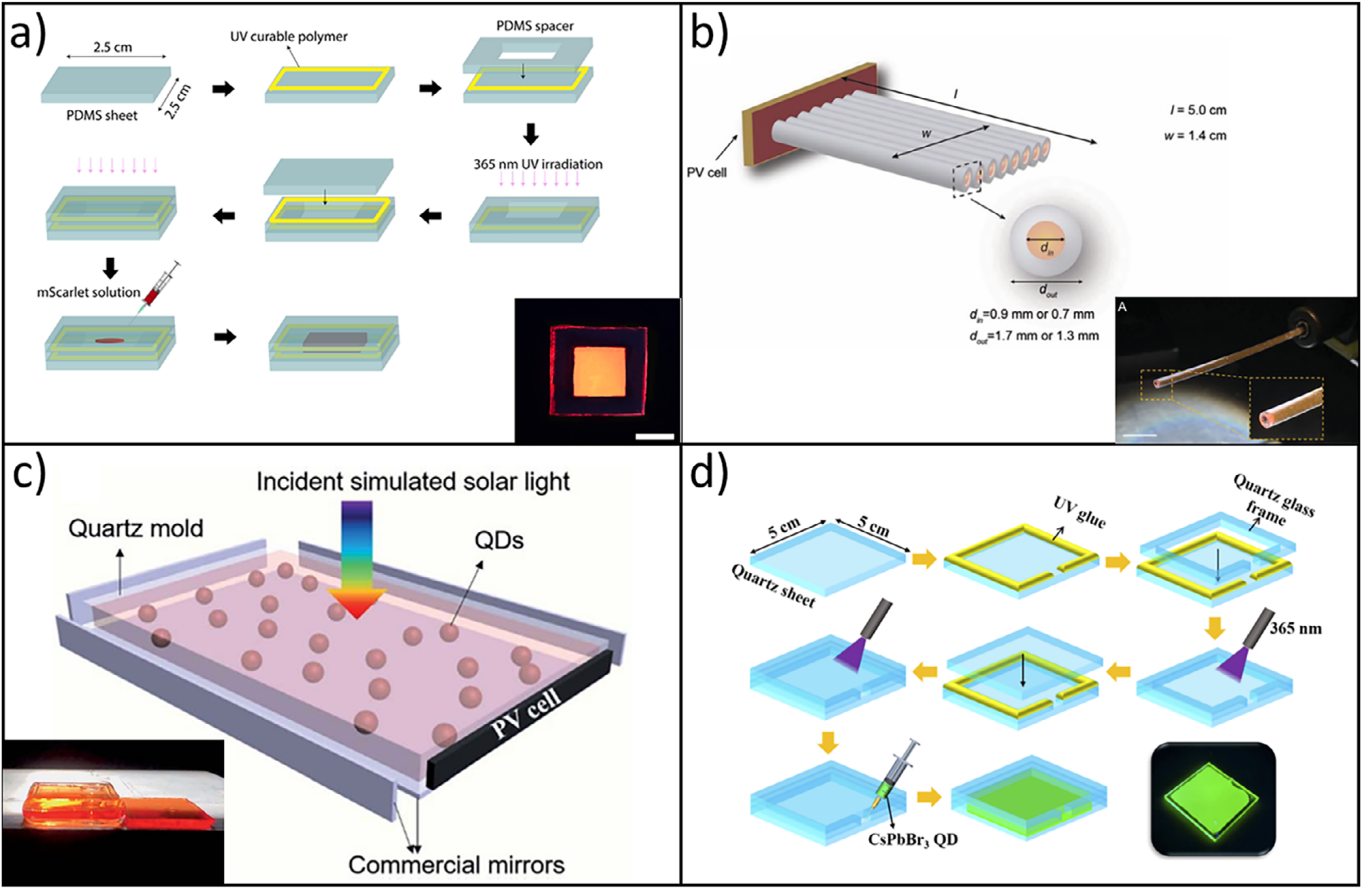
a) Schematic of the fabrication process for liquid LSCs. Reproduced (Adapted) with permission.^[^ ^79^ ^]^ Copyright 2019, ACS. b) Scheme of the fiber c‐LSCs bundle structure. Reproduced (Adapted) with permission.^[^ ^42^ ^]^ Copyright 2019, Wiley. c) Schemes of optical evaluation of different LSCs integrated with a PV cell of liquid LSC. Reproduced (Adapted) with permission.^[^ ^43^ ^]^ Copyright 2020, RSC. d) Schematic of the fabrication process of CsPbBr_3_ QD‐based liquid LSC. Reproduced (Adapted) with permission.^[^ ^83^ ^]^ Copyright 2022, Optica.

A device design method is reported in **Figure** **6**a, used by Sadeghi et al., in which a PDMS slab (2.5 × 2.5 × 0.2 cm^3^) was fabricated using an aluminum mold. A PDMS spacer was placed atop the base sheet to define the liquid chamber, and UV‐curable polymer was applied at the edges for sealing. After UV curing, a second PDMS sheet was added to enclose the structure, forming a central hollow cavity. A 5 wt% mScarlet protein solution (OD = 0.0588 at 500 nm) was injected into the cavity using a micro syringe.^[^ ^79^ ^]^ Leveraging the R‐PE solution's ability to absorb AM1.5G solar radiation and re‐emit in the visible range, it was incorporated into hollow‐core polymer optical fibers (POFs) (Figure 6b). This enabled the fabrication of liquid‐based cylindrical LSCs (c‐LSCs) using natural dyes. The development of these short‐length devices demonstrates the potential for scalable, flexible, and transparent c‐LSCs through bundled (PMMA)‐optical fibers (POF) assemblies filled with R‐PE solution. A semi‐industrial optical fiber manufacturing facility^[^ ^87^ ^]^ was used to fabricate the POFs, which were cut into lengths of ≈5 × 10^−2^ m. The hollow cores were filled with R‐PE solutions using a syringe. One end of each fiber was sealed with NOA68 resin to couple it with the PV device, while the opposite end was kept open. The fibers were then assembled into bundles.^[^ ^79^ ^]^

As shown in Figure 6c, the device design incorporated a liquid waveguide apparatus that used a rectangular quartz mold with a width of 20 mm and an inner height of 2 mm. The length of the mold varied depending on the desired geometry factors. QDs, with concentrations of up to 1.04 mg mL^−1^, were injected into the quartz waveguides through a small aperture with a syringe. The mold was subjected to UV radiation (360 nm) for 2 h, leading to the formation of liquid LSCs. Commercial mirrors were then applied to the edges and bottom of the two different LSCs to reflect light. In another study, Li et al. used a quartz glass mold that was fabricated for the CsPbBr_3_ QDs‐based liquid LSC in Figure 6d. A 5 cm × 5 cm × 0.2 cm quartz glass sheet served as the substrate, with UV glue applied to its edges to prevent leakage. A 4.5 cm × 4.5 cm × 0.1 cm quartz frame with a 2 mm injection port was placed on top and sealed under UV light. Another identical quartz sheet was then attached, forming a sealed cavity. And then solar cells were attached to the four edges to measure the photo‐electrical property.^[^ ^85^ ^]^

A critical aspect in the advancement of liquid LSCs is the meticulous design and configuration of the device, particularly with regard to ensuring structural integrity and operational stability. One of the major challenges associated with liquid LSCs is the risk of contamination and leakage of the luminescent liquid medium, which can significantly compromise both the performance and longevity of the device. To address this, a robust and leak‐proof containment system must be developed, typically in the form of a sealed cuvette, chamber, or box, which can effectively prevent the external contaminants and the escape of the active liquid. Designing such a container at the laboratory scale is inherently complex and requires precise engineering, material compatibility assessments, and attention to sealing techniques to ensure that the system remains closed and stable over time. These practical limitations often hinder broader experimental investigations and contribute to the relatively limited scope of research in this area. Therefore, before optimizing optical properties or scaling the device for real‐world applications, the primary and most fundamental step is the successful fabrication of a reliable, contamination‐resistant, and leak‐proof liquid LSC housing.

### 3.5 Leak‐Proof Containment: Design, Failure Modes, and Testing Protocols

Ensuring leak‐proof containment is a critical requirement for the long‐term stability of liquid LSCs, yet it has often been addressed only at a qualitative level. A systematic framework can instead be built around three pillars: design optimization, failure mode analysis, and standardized testing.

From a design optimization perspective, both chamber geometry and host material selection dictate evaporation resistance and long‐term structural integrity. As shown by Liu et al., temperature fluctuations between 10 and 40 °C significantly affect efficiency and stability in polymeric LSCs, highlighting that glass or quartz chambers provide superior barrier performance compared to flexible PDMS hosts.^[^ ^88^ ^]^ Recent studies on host matrix engineering demonstrate that the use of UV‐curable polymers can significantly improve the optical stability and durability of LSCs, particularly in thin‐film configurations. Complementary reviews further emphasize that matrix innovations such as fluoropolymers and other advanced polymeric hosts play a pivotal role in balancing optical clarity with long‐term stability. From a design optimization perspective, these findings suggest that for liquid LSCs, the careful selection of matrix and sealing materials is equally critical as waveguide geometry, since both optical efficiency and containment robustness ultimately govern device performance.^[^ ^89^, ^90^ ^]^

Failure mode analyses across LSCs consistently identify solvent loss, photodegradation, and polymer permeability as the dominant risks. Slooff et al. demonstrated that polymer matrices are prone to photo‐oxidation and thermal cracking under extended outdoor illumination, leading to scattering and reduced waveguiding.^[^ ^91^ ^]^ More recently, long‐term durability assessments of liquid and solid LSCs confirmed that solvent evaporation, if not mitigated, remains a key degradation pathway. For example, even a small injection aperture can lead to ≈5% mass loss under moderate heating, underscoring the vulnerability of weakly sealed designs.^[^ ^92^ ^]^ Collectively, these findings frame leakage and photo‐chemical instability not as incidental lab issues, but as systematic failure modes that must be anticipated and engineered against.

Finally, testing protocols are essential for establishing reproducible benchmarks of stability. Here, the LSC community can draw from encapsulation practices in both photovoltaics and large‐volume liquid detectors. For instance, the Daya Bay antineutrino detector program implemented rigorous leakage tests on multi‐liter stainless‐steel vessels using vacuum, pressure, and tracer methods to guarantee long‐term containment.^[^ ^93^ ^]^ Similar gravimetric, thermal‐cycling, and accelerated UV protocols are directly transferable to liquid LSC housings, enabling systematic screening of sealant performance and device integrity. By adopting such standardized reliability assessments (e.g., ASTM F2096 bubble testing, ISO thermal cycling protocols), LSC research can move from qualitative claims of “stability” toward verifiable engineering validation.

## 4 Main Advantages of Liquid LSCs over Thin‐Film LSCs

Liquid LSCs offer several advantages over traditional solid‐state or thin‐film LSCs, especially when considering flexibility, tunability, scalability, and potential for sustainable design. Below is a detailed breakdown of what can be saved or gained with liquid LSCs, along with their key advantages:

### 4.1 Reconfigurability and Design Flexibility

Liquid LSC offer significant advantages in reconfigurability and design flexibility due to their fluidic active medium. Reconfigurability refers to the ability to easily modify or adapt the device by changing its fluidic active medium. Unlike solid‐state LSCs, the liquid medium can be drained, replaced, or adjusted in real time, enabling quick tuning of optical properties, repair, or testing of new luminophores without altering the physical structure. This allows for dynamic tuning of absorption and emission spectra, PLQY, and Stokes shift, such as adjusting spectral response or switching transparency/color by modifying the luminophore concentration or incorporating responsive materials within the liquid.^[^ ^85^ ^]^ Beyond reconfigurability, the inherent fluidity provides exceptional design flexibility, allowing liquid LSCs to be integrated into diverse and complex geometries like curved surfaces or tubular structures, offering aesthetic and architectural versatility that is challenging for rigid thin‐film LSCs.^[^ ^94^ ^]^ This opens up possibilities for multifunctional designs, where the LSC can serve not only for energy generation but also for dynamic shading, privacy control, or artistic installations in BIPV applications.^[^ ^43^ ^]^

### 4.2 Cost Efficiency and Material Reusability

Liquid LSCs offer distinct advantages in terms of cost efficiency and material reusability that extend beyond just the active luminophore material to encompass the entire device structure. A primary driver of cost in LSCs is the luminophore material, which can be expensive, especially for highly efficient QDs or organic dyes. In thin‐film LSCs, these luminophores are permanently embedded within a solid polymer matrix, making their recovery and reuse challenging and often uneconomical at the end of the device's lifespan. This results in a “take‐make‐dispose” model for these valuable materials.^[^ ^95^ ^]^ In contrast, liquid LSCs allow for the recovery, purification, and reuse of the expensive luminophores from the solution when the LSC reaches its end of life or if the luminophores degrade.^[^ ^43^ ^]^ Crucially, the transparent containment structure (e.g., glass or polymer sheets) of a liquid LSC can also be readily cleaned and refilled with fresh luminescent solution, or repurposed entirely for other applications, significantly extending its useful life and minimizing waste generation. Moreover, the host solvents (e.g., water, ionic liquids, or ethanol) utilized in liquid LSCs are often considerably cheaper than the specialized solid polymer matrices required for thin‐film LSCs, contributing to lower initial fabrication costs. This combined reusability of both the active material and the passive structural components, along with the use of more affordable solvents, drastically reduces the long‐term material cost, as the same active components can be employed in new LSC devices, and the expensive housing can be reused, thereby decreasing the reliance on virgin materials and mitigating supply chain risks associated with rare or specialty chemicals. This leads to potential for a significantly lower lifecycle cost due to the inherently refillable and recyclable design. Furthermore, the ability to replenish degraded luminophores in liquid LSCs directly translates to a longer effective lifespan of the overall LSC device and its optical components, further enhancing its cost‐effectiveness over time by delaying the need for full replacement. This closed‐loop approach to material management aligns with principles of circular economy, offering a more sustainable and economically viable solution for widespread adoption of LSC technology.^[^ ^42^, ^85^ ^]^

### 4.3 Homogeneous Dispersion and Enhanced Optical Quality

The ability of a liquid medium to facilitate superior dispersion of active luminescent materials is a critical factor in achieving higher LSC performance. Dyes, CDs, or QDs can be more uniformly dispersed in liquids than in solid polymer matrices. In solid polymers, there is an inherent tendency for luminophores to aggregate, especially at higher concentrations required for efficient light harvesting.^[^ ^96^ ^]^ This aggregation leads to self‐quenching, where the proximity of luminophore molecules causes nonradiative energy transfer and a reduction in emitted light. In a liquid solvent, the luminophores are free to move and remain well‐separated, minimizing these aggregation‐related quenching effects.^[^ ^43^ ^]^ This superior dispersion results in a significantly higher PLQY, meaning a greater percentage of absorbed photons are re‐emitted as useful light. Furthermore, the homogeneous distribution of luminophores in a liquid reduces local variations in refractive index and particle clustering, which are common sources of scattering losses in solid LSCs.^[^ ^97^ ^]^ By minimizing these scattering events, more of the re‐emitted light is effectively guided towards the edges of the LSC for collection by photovoltaic cells, leading to fewer scattering losses and ultimately enhancing the overall optical efficiency and power conversion efficiency of the LSC device.

### 4.4 Larger Stokes Shift and Tunable Optical Properties

The Stokes shift is vital in LSCs because a larger shift reduces the spectral overlap between absorption and emission, thereby minimizing self‐absorption losses (where emitted light is re‐absorbed by other luminophore molecules).^[^ ^98^ ^]^ In liquid LSCs, it is significantly easier to tune solvent polarity or pH, which are well‐known parameters that can strongly influence the electronic transitions within many organic dyes and QDs. This tunability allows for fine‐tuning of the luminophore's emission spectrum, effectively increasing its Stokes shift and thereby reducing reabsorption losses.^[^ ^85^ ^]^ Furthermore, the liquid medium provides an ideal environment for precisely controlling the arrangement and proximity of different luminescent species. This enables the sophisticated combination of multiple dyes using Förster resonance energy transfer (FRET) mechanisms in a controlled manner to optimize energy transfer and spectral coverage.^[^ ^99^ ^]^ By strategically selecting dyes with overlapping absorption/emission bands and appropriate energy levels, energy can be efficiently transferred from higher‐energy absorbing dyes to lower‐energy emitting dyes, funneling collected light towards the photovoltaic cells with minimal internal losses and broadening the effective spectral range of solar light capture. This level of precise spectral engineering and control over intermolecular interactions is far more challenging to achieve in rigid solid polymer matrices.

### 4.5 Easier Integration with Large and Irregular Surfaces

Unlike rigid thin‐film LSCs that are typically limited to flat or slightly curved substrates, liquid LSCs can be embedded in highly flexible or complex curved geometries.^[^ ^85^ ^]^ This is because the liquid luminophore solution conforms to the shape of its transparent containment structure, which can be custom fabricated from materials like flexible polymers or intricately shaped glass. This inherent adaptability makes them exceptionally suitable for BIPV in a wide range of scenarios, including curved windows, aesthetically diverse building facades, and even the irregular surfaces of greenhouses or skylights.^[^ ^94^ ^]^ Their ability to fill non‐planar spaces allows for a more seamless and less obtrusive integration of solar energy harvesting into architectural designs, enabling both energy generation and simultaneous functionalities like dynamic shading or enhanced daylighting, which is crucial for the broader acceptance and deployment of solar technology in urban environments.^[^ ^98^ ^]^

### 4.6 Better Thermal Management

As LSCs absorb sunlight, a portion of the energy is inevitably converted into heat, particularly by non‐radiative recombination processes within the fluorophores and by reabsorption of emitted photons. In solid polymer matrices, this accumulated heat can lead to elevated temperatures within the device, accelerating the thermal degradation of the embedded fluorophores, thereby reducing their PLQY and shortening the LSC's operational lifespan.^[^ ^100^ ^]^ Furthermore, this inherent thermal management capability facilitates thermal regulation in integrated PV systems, where LSCs are often coupled with solar cells. By keeping the LSC and, by extension, the attached PV cells cooler, the overall efficiency of the combined system can be maintained at higher levels, as PV efficiency typically decreases with increasing temperature, making liquid LSCs a promising solution for long‐term, high‐performance solar energy harvesting.

### 4.7 Scalability and Fabrication Simplicity

The fabrication of thin‐film LSCs often requires sophisticated deposition techniques, such as spin‐coating, dip‐coating, or vapor deposition, to create uniform and defect‐free films. In contrast, liquid LSCs can be easily prepared by dissolving or dispersing luminescent materials into a suitable solvent and encapsulating them in transparent containers made of glass or polymer.^[^ ^42^, ^43^ ^]^ This simplified preparation not only lowers the fabrication cost but also allows for easy maintenance and rejuvenation, the luminescent solution can be replaced or refreshed if degradation occurs, effectively extending the device's operational lifetime. Additionally, liquid LSCs are more readily scalable, since they can be implemented using simple, inexpensive manufacturing processes such as mold casting or container filling, without requiring cleanroom conditions or advanced fabrication equipment.^[^ ^79^, ^85^ ^]^

## 5 Applications for Liquid LSC Technology

Liquid LSCs promise tunable spectra, and thermal dissipation advantages compared to solid slabs, but practical use hinges on how well liquid‐specific issues leakage/encapsulation, solvent volatility oxygen ingress, photostability, and thermal expansion are managed for each application class.

### 5.1 Building‐Integrated Photovoltaics

For window/façade integration, requirements include multi‐year outdoor durability, edge‐illumination‐compatible module designs, and compliance with building/PV standards (leakage safety, flammability classes, pressure cycling). A recent BIPV‐focused LSC review quantifies feasible target PCEs for semi‐transparent glazing (≈4.5%–5% at commercial scale with ≈50% visible transmission) and stresses scale‐up losses and certification barriers, particularly relevant for liquids that must remain sealed long‐term. Year‐long outdoor data on large LSCs (QD‐based) document spectral drift and efficiency changes under real weathering, underscoring the need for robust sealing and maintainable designs before architectural adoption.^[^ ^31^ ^]^

Key thresholds: leak‐free, UV/thermal/humidity‐stable liquids and seals over decades; visible transmission per glazing class; PCE approaching targets.^[^ ^31^ ^]^

### 5.2 Wearables and Portable Power

For body‐proximal or handheld use, shorter lifetimes (≈2–5 years) are acceptable, but safety (low‐toxicity solvents, robust containment) and mechanical flexibility dominate. Liquid‐enabled formats hollow‐core/liquid‐core waveguides and refillable reservoirs provide serviceability and light, flexible geometries suitable for garments or portable chargers.^[^ ^42^, ^85^, ^101^ ^]^ Recent high‐efficiency liquid LSCs (optimized spectra/coupling) show that when liquids are well contained, meaningful power densities are achievable at device scale.^[^ ^85^ ^]^

Key thresholds: PLQY retention under thermal cycling; skin‐safe or encapsulated solvents; impact‐resistant, leak‐proof packaging.

### 5.3 Agrivoltaics and Greenhouses

Greenhouses benefit from spectral selectivity (absorb UV/blue; transmit PAR 400–700 nm), non‐toxic, food‐safe liquids, and humidity‐resistant sealing. Field and modeling studies on LSC greenhouses show the potential for co‐generation while maintaining crop‐relevant transmission; recent work explores CQD luminophores tailored for transparent greenhouse covers.^[^ ^36^, ^102^ ^]^ For liquids, contamination control and seal integrity are critical to prevent solvent ingress into growing spaces.

Key thresholds: PAR transmission targets for crop yield; verified nontoxicity; 10‐year optical/seal stability in humid conditions.

### 5.4 Off‐Grid/Remote Power and Humanitarian Use

Refillable liquid‐fiber or modular reservoir LSCs can trade lifetime for serviceability: periodic fluid replacement can reset optical performance without discarding the module. Demonstrations of liquid‐filled hollow‐core fiber bundles and compact liquid devices support this pathway.^[^ ^42^, ^102^, ^103^ ^]^

Key thresholds: simple field‐refill procedures; stable PLQY under temperature swings; rugged housings.

### 5.5 Photodetectors and Light‐Sensing

Liquid matrices enable tunable, high‐gain waveguides and sensing (e.g., strain/illumination) where precision matters more than 20‐year lifetimes. Liquid‐core polymer optical fibers show simultaneous light conversion and sensing with large flexibility windows well‐matched to low‐power, embedded sensors rather than energy generation.^[^ ^101^ ^]^

## 6 Comparative Analysis of Liquid and Solid/Thin Films

Both thin‐film/solid‐state and liquid LSCs operate on the same optical principle of photon absorption, re‐emission, and waveguiding toward edge‐mounted PV cells, but their performance trade‐offs differ substantially.

### 6.1 Solid‐State/Thin‐Film LSCs

Typically based on polymer hosts such as PMMA or PVB doped with organic dyes (e.g., Lumogen F Red 305, Rhodamine 6G) or quantum dots are comparatively mature, offering excellent mechanical durability, compatibility with standard lamination techniques, and closer alignment with BIPV certification pathways.^[^ ^30^, ^104^ ^]^ However, they face intrinsic limitations such as irreversible photo‐oxidation of dyes, constrained loading concentrations due to aggregation, and limited thermal management since polymers trap heat.

### 6.2 Liquid LSCs

In contrast, provide unique strengths: continuously tunable absorption/emission by varying luminophore concentration or solvent refractive index; enhanced heat dissipation due to liquid convection; and ease of reconfiguration, since liquids can be replaced or replenished to restore performance. These advantages, however, come with drawbacks that solid counterparts largely avoid: leakage and evaporation risks require advanced containment designs^[^ ^88^ ^]^ contamination (oxygen, moisture, dust) directly degrades PLQY in solution; and mechanical integration challenges as liquid modules need rigid encapsulation and sealing to function in outdoor BIPV or portable contexts.

From a performance perspective, state‐of‐the‐art solid‐state LSCs routinely achieve external photon efficiencies (η_ext_) in the 5%–7% range for >100 cm^2^ devices,^[^ ^81^, ^105^ ^]^ while liquid systems remain at 2%–4% for similar scales^[^ ^71^ ^]^ with degradation over time as an additional bottleneck. This gap is not purely due to materials but reflects the lack of standardized protocols, solvent‐induced loss mechanisms, and sealing limitations. Unlike solid‐state LSCs, which typically rely on Lumogen F Red 305 in PMMA as a benchmark material for cross‐study comparison,^[^ ^81^, ^105^ ^]^ liquid LSCs currently lack such a standardized reference dye or system. This absence complicates benchmarking of efficiency data across studies.

In summary, liquids are not a direct replacement for solid‐state LSCs but rather a complementary platform: their tunability, thermal advantages, and serviceability could make them attractive for specialized applications (e.g., dynamic glazing, reconfigurable or niche PV), while solid‐state devices remain the frontrunner for near‐term commercialization

## 7 Challenges and Limitations

Despite their significant advantages, liquid LSCs face several challenges and limitations that must be addressed for widespread commercialization, particularly when compared to more established thin‐film LSC technologies. **Table** **3** provides a comparative overview of liquid and thin‐film LSCs, highlighting key performance metrics and sustainability‐related parameters reported in recent literature. One of the primary concerns is their limited chemical, thermal, and photochemical stability. The use of liquid media, often comprising organic solvents or aqueous solutions, makes them susceptible to evaporation, oxidation, and photodegradation, especially under continuous solar exposure. Certain solvents may degrade or react with the luminophores or the container itself, leading to reduced efficiency or leakage. Photochemical degradation of the luminophores, while potentially mitigated by some liquid environments, can still occur, and the long‐term stability of the overall liquid formulation under real‐world operating conditions remains a key research area. Another challenge involves reabsorption and scattering losses. Although liquid LSCs can achieve more homogeneous dispersion than solid films, which reduces aggregation‐related quenching, achieving optimal light guiding efficiency across large areas can still be hindered by inherent reabsorption if the Stokes shift is not sufficiently large or if the luminophore concentration is too high. Moreover, while liquids can inherently offer clearer media, any particulate impurities or long‐term instability that leads to phase separation or precipitation within the liquid can introduce new scattering centers, diminishing optical quality. Finally, scalability and manufacturability present practical hurdles. While the concept of simply filling a container with liquid seems straightforward, ensuring leak‐proof, durable, and cost‐effective large‐area encapsulation for decades of outdoor exposure is a significant engineering challenge. Manufacturing processes for precise filling and sealing large, potentially complex liquid LSC panels, especially for building‐integrated applications, need to be developed and proven for mass production. These factors can impact on the overall reliability and competitive cost of liquid LSCs compared to more mature thin‐film fabrication techniques.

**Table 3:** Comparison between liquid LSCs and Thin‐Film LSCs based on key performance and sustainability parameters.

Parameter	Liquid LSC	Thin‐film LSC
**PLQY**	Moderate to high (typically 30%–80% depending on fluorophore/dot concentration and solvent)	High (>70%–90%) due to controlled matrix environment reducing non‐radiative losses
**PCE**	Moderate (1%–3%). Efficiency depends on light management and PV coupling	Moderate (1%–3%). Better light guiding and stability lead to improved performance.
**Photostability**	Lower, susceptible to photobleaching, oxidation, or aggregation in solution	Higher, dopants are embedded in a stable polymer or glass matrix.
**Optical Clarity and Scalability**	Excellent transparency and scalability through fluidic systems or microchannels	Good, but fabrication can become complex at larger scales.
**End‐of‐Life Recycling Potential**	Highly active material can be recovered or replaced in fluidic systems	Moderate, embedded luminophores are harder to extract.
**System Flexibility & Tunability**	Very high, easy to tune optical properties by changing the fluid composition	Moderate, once fabricated, changing dopants or matrix requires refabrication
**Mechanical Stability**	Lower risk of leakage, evaporation, or degradation of solvent over time	Higher, solid‐state device with minimal maintenance

### 7.1 Life Cycle Assessment of Luminescent Materials for LSCs

While many studies qualitatively emphasize the sustainability of novel functional materials, recent research has increasingly adopted quantitative environmental impact assessments and life cycle methodologies to provide a more rigorous perspective. For example, Muteri et al. evaluated an innovative LSC photovoltaic smart window, reporting not only enhanced optical and thermal performance but also measurable environmental benefits. Their analysis demonstrated electricity generation of ≈10 Wh/month, combined with up to 50% reductions in heating and cooling demand compared to conventional glazing systems, thereby situating the technology within the EU's targets for greenhouse gas reduction and renewable energy uptake.^[^ ^33^ ^]^ Complementing this, Fernandes et al. performed a cradle‐to‐grave life cycle assessment (LCA) of six different CD synthesis routes, normalizing impacts per kilogram of product. Strikingly, their results showed that high‐yield syntheses (40.1% yield) were not inherently more sustainable, as the associated energy and chemical burdens often outweighed the benefits of increased yield. In fact, a microwave‐assisted “standard” route (28.5% yield) exhibited the lowest global warming potential and toxicity, demonstrating that process efficiency and precursor choice are more critical sustainability levers than yield alone.^[^ ^104^ ^]^ A companion study by the same group compared two high‐yield CD synthesis strategies, one based on hydrothermal + alkaline peroxide treatment of hydrochar, and another employing eutectic salt‐assisted thermal treatment. The former route was shown to have significantly lower environmental impacts, with the salt‐based method penalized by the production and disposal of salts. Sensitivity analysis further revealed that the carbon precursor identity is the dominant driver of impacts, with biomass‐derived precursors reducing global warming potential by up to 30% compared to petrochemical sources.^[^ ^106^ ^]^ The environmental implications of LSC smart windows have also been examined through full LCA. Muteri et al. quantified the global warming potential (100 years) of an LSC‐integrated smart window at 5.91 × 10^3^ kg CO_2_ eq, with manufacturing contributing ≈96% of the impact. Importantly, recycling scenarios reduced impacts by up to 45%, while using 75% recycled aluminum in the window frame cut emissions by 3%–46% depending on the impact category. Component‐level analysis identified the aluminum frame (≈60%) and shading system batteries/motors (≈30%–36% in abiotic depletion) as critical hotspots. When impacts were normalized per unit area, the LSC modules showed 870% lower impacts than conventional PV modules, but when normalized per kWh generated, their impacts were 200%–1900% higher, reflecting the trade‐off between multifunctionality and lower energy conversion efficiency.^[^ ^107^ ^]^ Finally, Jeevitha et al. extended LCA to QDs and their composites, highlighting that despite their promise as “green” alternatives, production remains energy‐ and reagent‐intensive, with GWP values in the range of 0.2–0.5 kg CO_2_ eq kWh^−1^ depending on synthesis pathway. Moreover, heavy‐metal‐based QDs such as cadmium and lead were shown to dominate toxicity‐related categories, emphasizing the importance of nontoxic precursors and energy‐efficient synthesis methods. Substitution with biomass precursors and adoption of microwave heating reduced impacts by more than 40% in toxicity categories, illustrating the potential for greener nanomaterial design.^[^ ^108^ ^]^

Together, these studies demonstrate that novel functional materials can indeed achieve quantifiable sustainability benefits, providing a solid foundation to support their classification as environmentally friendly. By documenting improvements in global warming potential, energy demand, recyclability, and toxicity as stated in **Table** **4**, they establish measurable evidence that these materials are not only high‐performing but also aligned with sustainability goals. This quantitative basis ensures that claims of “green” or “eco‐friendly” character are substantiated, reinforcing the case for their integration into future eco‐design strategies and sustainable technologies.

**Table 4:** Quantitative environmental impact assessment of luminescent solar concentrators.

Material / technology	Metric reported	Key results	Sustainability implication	Refs.
Photovoltaic LSC smart window prototype	Energy savings, electricity generation	≈10 Wh/month electricity; up to 50% reduction in heating/cooling energy demand	Demonstrates that smart windows can both generate energy and cut building operational emissions.	[33]
Carbon dots (6 synthesis routes)	Cradle‐to‐grave LCA; GWP, toxicity, water use	High‐yield synthesis (40.1%) not always greener; microwave‐assisted (28.5%) had lowest GWP and toxicity	Highlights that synthesis route, not yield alone, determines sustainability.	[104]
High‐yield carbon dots (two routes)	Cradle‐to‐gate LCA	Hydrothermal + peroxide route lower impacts; salt‐based route burdened by eutectic salts. Biomass precursors cut GWP by ≈30%.	Identifies cleaner high‐yield strategy and importance of precursor selection.	[106]
LSC integrated smart window (SW–LSC)	Full LCA (cradle‐to‐gate + EoL)	GWP = 5.91 × 10^3^ kg CO_2_‐eq; 96% from manufacturing; recycling reduces impacts by 45%; recycled Al frame cuts 3%–46%.	Provides quantifiable benchmarks; shows recyclability and material substitution are key for eco‐design.	[107]
Quantum dots (QDs) and composites	LCA across categories (GWP, toxicity, CED)	GWP = 0.2–0.5 kg CO_2_‐eq kWh^−1^; heavy‐metal QDs dominate toxicity; biomass precursors reduce toxicity by 40%+	Demonstrates need for non‐toxic precursors and energy‐efficient synthesis to realize “green” QDs.	[108]

### 7.2 The Need for Standardized Protocols in Liquid LSC Research

A persistent challenge in LSC research is the lack of standardized testing protocols, which complicates meaningful comparison across studies. For solid‐state LSCs, several groups have proposed best practices and consensus‐style checklists for characterization and reporting.^[^ ^81^, ^105^ ^]^ These emphasize reporting internal and external photon efficiencies (η_*int*_, η_*ext*_), device efficiency (η_*dev*_), geometric gain (*G*), and illumination conditions using standard AM1.5G spectra.^[^ ^109^ ^]^ However, protocols tailored specifically for liquid LSCs are still absent.

Liquid LSCs introduce additional sources of variability does not present in slab‐based systems such as solvent refractive index, fluorophore concentration in solution, meniscus scattering, oxygen quenching, and solvent evaporation. Without consistent reporting on these factors, reproducibility and cross‐study comparisons remain elusive. In the absence of formal standards, we recommend the field consider a preliminary, liquid‐specific set of reporting practices. Ideally, studies should report: (i) solvent identity, refractive index, and path length; (ii) dye or quantum dot type and concentration; (iii) container material and sealing method; (iv) absorption and emission spectra plus photoluminescence quantum yield, measured via integrating sphere (with a solvent blank); (v) η_*int*_ and η_*ext*_ under standardized AM1.5G, Class A/AAA illumination, using appropriate masking and dark backgrounds; and (vi) stability tests conducted under controlled atmosphere and temperature, with degradation tracked over time.

Recognizing that PLQY measurements in solution require particular care, it is helpful to reference established protocols. For instance, the IUPAC technical report by Brouwer emphasizes the need for reliable standard materials, careful absorbance matching, accurate spectral corrections, and awareness of inner‐filter effects in dilute solutions.^[^ ^110^ ^]^ Similarly, Fries et al. recommend multiple independent measurements to assess statistical uncertainty in PLQY determinations. By integrating such practices into liquid LSC characterization, we can enhance the rigor of reported results.^[^ ^111^ ^]^

While this suggested checklist is not intended as a prescriptive standard, it highlights critical parameters that and if uniformly reported would enable reproducible benchmarking across studies in the liquid LSC domain. We intend this as a starting point for broader community dialogue and refinement, analogous to prior consensus efforts in the LSC field.^[^ ^81^, ^105^ ^]^ Moving toward uniform characterization and transparent reporting will accelerate systematic progress in liquid LSC research.

### 7.3 Integration of Liquid LSCs with Photovoltaic Systems

Despite recent advances, several unresolved challenges continue to hinder the integration of liquid LSCs with PV systems. These barriers span mechanical stability, electrical coupling, and system‐level compatibility, and together they represent critical bottlenecks preventing transition from laboratory demonstrations to real‐world deployment.

#### 7.3.1 Electrical and Mechanical Challenges

Electrically, effective coupling requires precise spectral matching between the luminophore emission and the PV cell's EQE. A mismatch between the emission spectrum and PV absorption can lead to current imbalance particularly in series‐connected modules thereby reducing overall system efficiency. As highlighted by Sadeghi et al. and Jakubowski et al., the design of the liquid medium, whether implemented in bulk cells, fibers, or hybrid liquid–waveguide structures, directly determines the uniformity and intensity of illumination at PV edges. Proposed solutions such as index‐matching gels and anti‐reflection coatings can reduce coupling losses, but their long‐term durability remains uncertain because UV exposure and thermal cycling degrade their optical properties over time.^[^ ^79^, ^112^ ^]^

Mechanically, liquid LSCs are inherently less stable than solid‐state counterparts due to risks of solvent leakage, evaporation, and oxygen quenching. Zhang et al. demonstrated that laminating aqueous LSC layers between glass substrates can improve structural robustness, yet outdoor studies such as the one‐year quantum‐dot LSC test by de Bruin et al. revealed that stability and spectral integrity under real environmental conditions remain persistent challenges. In addition, thermal expansion of liquids within sealed modules introduces mechanical stresses that can compromise encapsulation. More recent work by Frías et al. explored serviceability improvements via glass‐encapsulated liquids and hollow‐core fibers, though these approaches add complexity and cost.^[^ ^42^, ^113^, ^114^ ^]^ Debije et al. further emphasized that interface losses at the liquid–glass boundary remain a critical bottleneck, underscoring the need for improved interface engineering.^[^ ^115^ ^]^

#### 7.3.2 System‐Level Performance and Compatibility

At the system level, liquid LSCs challenge the assumptions of conventional PV module design. Standard modules are optimized for direct, face‐on illumination, whereas LSCs deliver concentrated, lateral edge illumination. Richards and Howard noted that this fundamental mismatch necessitates rethinking PV architectures, potentially requiring hybrid concentrator–PV assemblies.^[^ ^30^ ^]^

Practical deployment also raises concerns of architectural integration. As shown by Mangherini et al. embedding liquid LSCs into ventilated façades introduces issues of thermal load, wind resistance, and maintenance. Liquids are particularly sensitive to temperature fluctuations, which can alter solvent refractive index, dye solubility, and stability, compounding reliability concerns. Finally, as Meinardi et al. emphasized, compliance with building and PV certification standards remains an unmet requirement. Without standardized testing protocols, liquid LSC–PV modules cannot undergo the certification processes necessary for large‐scale adoption.^[^ ^32^, ^101^ ^]^

Unless these bottlenecks are systematically addressed, liquid LSCs will remain confined to laboratory studies. Overcoming them represents a crucial prerequisite for translating liquid LSC technology into deployable solar solutions.

### 7.4 Multiple Luminophores in Liquid LSCs: A Limitation and Pathway Forward

Single‐emitter liquid LSCs capture only a narrow solar band and are highly prone to reabsorption. Multi‐luminophore strategies such as dye–dye cascades and QD→dye hybrids—offer broader spectral coverage (UV→vis→NIR) and red‐shifted emission, reducing reabsorption losses before photons reach edge‐mounted PVs. However, in liquids, donor–acceptor behavior is strongly influenced by solvent polarity, viscosity, refractive index, and concentration, making reproducibility difficult and introducing risks of aggregation, oxygen quenching, and solvent evaporation.

Multi‐dye liquid LSCs typically rely on FRET to channel energy efficiently. The classical efficiency expression:

(11) $$E=1/(1+{\left(\frac{r}{R0}\right)}^{6}$$

shows dependence on donor–acceptor distance “r”, Förster radius “R0”​, spectral overlap, and donor PLQY. In liquids, donor–acceptor separation is determined by solution concentration, but high loading often triggers self‐quenching and aggregation. Tummeltshammer et al. modeled QD → dye cascades and demonstrated that FRET by linking QDs and dye molecules enhanced the optical efficiency of LSCs by at least 24.7% experimentally, with a theoretical maximum efficiency of 75.1% from simulations, an increase of over 215% compared to QD‐only systems.^[^ ^116^ ^]^ Lyu et al. optimized Förster energy transfer between a PDI‐Sil donor and a p‐O‐TPE acceptor in a polymer matrix to achieve high FRET efficiency with robust performance.^[^ ^117^ ^]^ Bailey et al. showed that using three BODIPY dyes in cascade within a polymer system increased relative light output significantly compared to single‐dye counterparts.^[^ ^118^ ^]^ These studies illustrate the potential of cascaded systems but also reveal high variability and underscore the need for standardized testing protocols.

Stability remains the largest limitation. Mooney et al. showed that in multi‐dye cascades, donor dyes can exhibit up to ∼18× improved photostability, whereas the terminal acceptor dye degrades more rapidly (1.6–1.9× faster than alone) under extended illumination.^[^ ^119^ ^]^ Oxygen exposure accelerates bleaching, while solvent volatility and thermal effects further destabilize spectra. Liquid‐crystal hosts show improved cycling stability but still face long‐term drift.^[^ ^120^ ^]^ To facilitate comparison across the literature, **Table** **5** summarizes representative studies on liquid LSCs. For each case, the table specifies the host type used, the experimental or modelling techniques adopted, and the principal findings reported. This comparative presentation highlights both the diversity of liquid‐based architectures and the recurring challenges and opportunities identified, thereby offering readers a concise reference point for evaluating progress in the field.

**Table 5:** Multi‐luminophore LSC studies (liquid‐focused).

Host type	Donor/acceptor	Key findings	Refs.
Modeled QD–dye system	QD → dye (linked)	Experimental FRET boosts η_opt_ by ≥24.7%; simulated max η_opt_ = 75.1% (>215% improvement over QD‐only)	[116]
Polymer cascade system	PDI‐Sil → p‐O‐TPE	High FRET efficiency with stable performance	[117]
Multi‐dye organic cascade	BODIPY dyes (3‐dye cascade)	Cascade increases light output relative to single dyes	[118]

## 8 Technical Roadmap for Overcoming Fundamental Limitations in Liquid LSCs

Liquid LSCs face obstacles rooted in the fluidic nature of the medium, namely long‐term photostability of dissolved luminophores, oxygen ingress and solvent volatility, leakage/encapsulation reliability, and thermal/mechanical stresses. A systematic roadmap tailored to liquid systems can be organized across three levels: materials, device engineering, and system integration.

### 8.1 Material‐Level Strategies

At the material level, the solvent and luminophore environment are central to liquid LSC stability. Conventional organic solvents suffer from volatility and photodegradation, but ionic liquid formulations have been shown to suppress evaporation and improve photostability, making them promising hosts for sustainable liquid LSCs^[^ ^42^ ^]^. Oxygen quenching is a uniquely liquid‐based degradation pathway, since oxygen diffuses freely into solution; incorporation of dissolved scavengers can mitigate this and significantly extend dye lifetime.^[^ ^120^ ^]^ In addition, structured liquid hosts such as liquid crystals provide temperature‐ and phase‐responsive control of Förster energy transfer, while reducing scattering losses compared to isotropic solvents.^[^ ^121^ ^]^ Microemulsions also offer liquid‐specific benefits by stabilizing nanocrystals and dyes while maintaining optical clarity, thereby suppressing aggregation and self‐quenching effects.^[^ ^122^ ^]^

### 8.2 Device and Engineering‐Level Approaches

At the device scale, containment and optical management are critical for liquid systems, since leakage, evaporation, and thermal expansion pose risks not encountered in solids. Encapsulation strategies based on glass lamination with chemically resistant seals have proven effective at containing liquids while preserving transparency.^[^ ^123^ ^]^ Alternative liquid‐specific architectures such as liquid‐core polymer optical fibers provide a waveguiding geometry with robust containment and serviceability.^[^ ^112^ ^]^ However, thermal expansion and heating remain pressing issues. Experimental studies have confirmed that quantum‐dot liquid LSCs experience PLQY degradation and spectral drift under elevated temperatures, underscoring the need for integrated thermal management in liquid module designs.^[^ ^88^ ^]^

### 8.3 System‐Level Strategies

At the system level, durability and certification represent the final barrier to deployment. Long‐term outdoor testing of quantum‐dot liquid LSCs has revealed spectral drift and reduced yield over a one‐year period, directly implicating solvent degradation and sealing failures in real‐world conditions.^[^ ^114^ ^]^ Liquid LSCs also introduce unique challenges in building integration, where leakage, flammability, and pressure build‐up under thermal cycling must be addressed through tailored standards. Recent reviews emphasize that building‐integrated PV modules are not designed for lateral edge illumination, so adapted certification pathways and design standards will be essential for real‐world adoption of liquid LSC modules.^[^ ^30^ ^]^

## 9 Conclusion

In conclusion, liquid LSCs have demonstrated significant potential as an alternative to conventional solid‐state and thin‐film LSCs for next‐generation solar energy applications. Their unique advantages, including tunability, solution processability, ease of large‐area fabrication, self‐healing capability, and potential for material reuse, make them particularly attractive for applications requiring flexibility, recyclability, and cost‐effective scalability. This review has highlighted how liquid LSCs can address several limitations faced by thin‐film systems, such as restricted surface coverage, limited reconfigurability, and fabrication complexity. Despite these advantages, research on liquid LSCs remains in their early stages, with challenges such as long‐term stability, leakage, and contamination still limiting their practical deployment. Nonetheless, recent advances in luminescent materials, host matrices, and device designs offer a promising outlook. Continued research focused on optimizing stability, improving PLQY, and developing robust containment systems will be essential to unlock the full potential of liquid LSCs in sustainable energy systems. As the field advances, liquid LSCs are poised to play a vital role in the development of dynamic, efficient, and circular solar harvesting technologies.

### 9.1 Emerging Trends and Future Perspectives

Liquid LSCs offer several advantages that make them attractive for next‐generation solar energy systems. Their tunable optical properties and ease of incorporating multiple fluorophores enable broad‐spectrum light harvesting, while the fluidic nature allows for self‐healing and dynamic adaptability. Liquid LSCs are lightweight, cost‐effective, and scalable, making them ideal for large‐area applications like windows or facades. Their flexible design also supports aesthetic integration into buildings, combining functionality with architectural appeal. Furthermore, the liquid LSCs are recyclable due to their readily variable concentration and replaceable luminophores in the same waveguides, which may present a viable route towards the development of sustainable and effective LSCs for more cost‐effective and ecologically friendly PV energy conversion. While liquid LSCs present many promising features, they are currently limited by issues including reabsorption loss, where emitted photons are reabsorbed by the luminescent species before reaching the photovoltaic cells, thereby reducing overall efficiency. Material stability poses another significant challenge; many luminescent materials degrade under prolonged exposure to sunlight, affecting the longevity and performance of LSCs. In addition, liquid LSCs are particularly vulnerable to leakage and contamination issues. Over time, evaporation, sealant degradation, or microfractures in the container can lead to fluid leakage, compromising device functionality and safety. Moreover, liquid medium can be susceptible to contamination by dust, microorganisms, or chemical impurities, which may quench photoluminescence or alter optical properties. Looking ahead, the integration of liquid LSCs with next‐generation photovoltaics, smart surfaces, and transparent solar technologies holds significant potential for transforming energy harvesting solutions. If these challenges can be effectively addressed, liquid LSCs could emerge as a key player in the future of renewable energy, providing scalable, efficient, and aesthetically adaptable solutions for sustainable power generation.

## Conflict of Interest

The authors declare no conflict of interest.
